# Identification and Detection of a Peptide Biomarker and Its Enantiomer by Nanopore

**DOI:** 10.1021/acscentsci.4c00020

**Published:** 2024-05-03

**Authors:** Laura Ratinho, Laurent Bacri, Bénédicte Thiebot, Benjamin Cressiot, Juan Pelta

**Affiliations:** † Université Paris-Saclay, Univ Evry, CY Cergy Paris Université, CNRS, LAMBE, 95000, Cergy, France; ‡ Université Paris-Saclay, Univ Evry, CY Cergy Paris Université, CNRS, LAMBE, 91025, Evry-Courcouronnes, France

## Abstract

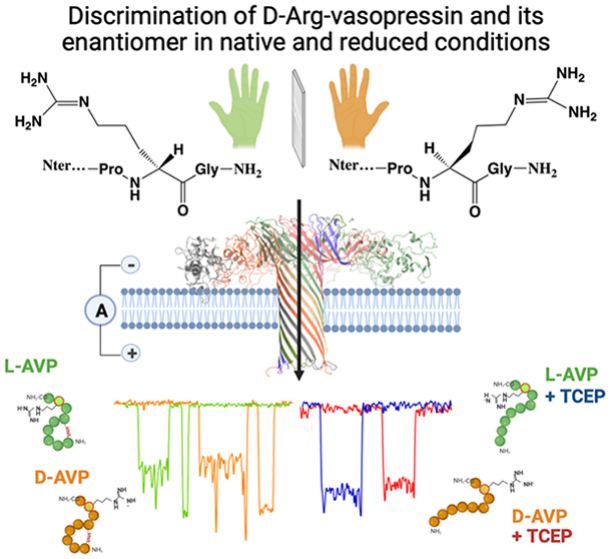

Until now, no fast, low-cost, and direct technique exists to identify and detect protein/peptide enantiomers, because their mass and charge are identical. They are essential since l- and d-protein enantiomers have different biological activities due to their unique conformations. Enantiomers have potential for diagnostic purposes for several diseases or normal bodily functions but have yet to be utilized. This work uses an aerolysin nanopore and electrical detection to identify vasopressin enantiomers, l-AVP and d-AVP, associated with different biological processes and pathologies. We show their identification according to their conformations, in either native or reducing conditions, using their specific electrical signature. To improve their identification, we used a principal component analysis approach to define the most relevant electrical parameters for their identification. Finally, we used the Monte Carlo prediction to assign each event type to a specific l- or d-AVP enantiomer.

## Abstract 2

An aerolysin nanopore allows the identification of vasopressin enantiomers as well as their conformation. A Monte Carlo prediction assigns each event type to a specific l- or d-AVP enantiomer.

## Introduction

l-Amino acid enantiomers are the predominant forms found in animal tissues. However, although d-amino acids are present in small quantities, they can be detected in either their free forms or polypeptide chains.^1^ In humans, the free d-amino acids, such as d-Asp, d-Ser, and d-Ala, play significant roles in various physiological functions, such as the development and functioning of the central nervous system, reproduction, and hormone release.^2−4^ Altered regulation production of these isomers can have profound effects on organisms. For example, during the natural aging process, there is a decline in the level of d-serine.^5^ Conversely, in pathological aging conditions, such as Alzheimer’s disease, there is excessive activation of serine racemase, resulting in hyperstimulation of the NDMA receptor due to an excess of d-Ser.^5^ Notably, levels of d-Arg levels are decreased.^6^

Additionally, several d-amino acids containing peptides (DAACPs) have been identified in various pathological conditions associated with diseases or natural aging processes, as evidenced by the presence of d-β-Asp-containing peptides in elastic fibers of sun-damaged skin,^7^d-Asp in αA-crystallin from the lenses of individuals with cataracts,^8^ and the β-amyloid peptide of Alzheimer’s patients.^9^ These DAACPs are mainly formed following the post-translational modification of l-amino acids, either by enzymatic racemization of l-amino acids into their d-form or spontaneously, despite a very slow process, within long-lived proteins and tissues.^1^ It has been established that oxidative stress and free radicals can induce such modifications in proteins.^10,11^ Notably, the alteration of a specific residue from l- to d-configuration often leads to a modification of the biological activity of the peptide and could be developed as therapeutic molecules.^12^ Modifications of peptide functional properties are likely due to a change in the higher-order structure of a protein.^13,14^ The significant increase of DAACPs discovered in diseases has encouraged researchers to consider them as potential biomarkers,^1^ and any new technique capable of detecting/quantifying or finding new DAACPs, even at low concentrations, would represent a major advancement in early diagnosis.

As mentioned above, there is a clear need to develop fast, low-cost, and direct techniques to identify chiral amino acids in a polypeptide chain without prechemical/enzymatic treatment. Due to the difference between l- and d-amino acids, we expect the polypeptide chains to adopt different conformations. Therefore, developing a technique that can sense the protein conformational landscape is necessary, while just a few studies have already been published using nanopores.^15−17^ To the best of our knowledge, the classical methods used, such as mass spectrometry, liquid chromatography, or enzymatic assays, for the detection of l- and d-amino acids do not allow the simultaneous detection of proteins, their conformation, and their chiral amino-acid composition.^14^

The nanopore electrical detection technique offers a unique multiscale analytical tool for peptide and protein enantiomer biomarker detection. Up to now, nanopores have been used to discriminate between chiral forms of free amino acids,^18^ as well as detect and identify protein/peptide biomarkers.^19−29^ More recently, research has focused on detecting post-translational modifications (PTMs)^21,30−37^ in peptide biomarkers that could help understand their implications in pathologies.^38−40^ Machine learning is now used to validate the data from the sequencing of individual amino acids^37^ to the identification of biomolecules^41,42^ and biomarkers.^43^

While the first study on detecting individual chiral amino acids (Trp, Phe, Tyr, Cys, and Asp) using an engineered Cu^2+^ phenanthroline alpha-hemolysin channel was published in 2012 by Bayley’s group,^18^ only a few studies have been published since. Two other groups showed the ability to discriminate individual l- and d-amino acids using cyclodextrin adapters^44^ inside a covalent organic framework-(His) or an alpha-hemolysin mutant^45^ (Phe, Trp, Tyr). Mubarak et al. developed a nanopipette system to detect single amino-acid enantiomers (Tyr, Trp, and Phe) using a polymeric conical nanopore functionalized with BSA.^46^ They used current rectification to detect each kind of enantiomer. The first example of detecting chiral amino acids within a polypeptide biomarker was described by Luchian’s group, where they identified, using Cu^2+^ chiral recognition, the presence of histidine enantiomers.^47^ A recent study used mutant OmpF to control the side chain orientation of a β-amyloid peptide inside the nanopore due to its lateral electric field.^48^ They were able to discriminate between d-Ser and d-Asp isoforms and mutants. Finally, Maglia’s group studied the detection of d-Ala and d-Leu in enkephalin peptides using FraC and CytK mutants.^49^

Until now, no studies have been conducted to detect enantiomers within a biologically relevant peptide that has secondary and tertiary structures such as disulfide bonds. Furthermore, most previous studies needed an adapter or several pore mutations to detect chiral amino acids in the polypeptide sequence. To prove the ability to sense enantiomers containing elements in secondary and tertiary structures under native conditions, we used vasopressin as a model peptide. Vasopressin (l-Arg-AVP, later named l-AVP), a hormone produced in mammals, is involved in the regulation of water balance and blood pressure, as well as having an influence on social behavior, memory, and the cardiovascular system through its interaction with V1a, V1b, and V2 receptors.^50^ Vasopressin quantification was performed to assess hyper- or hyposecretion pathologies. In the case of diabetes insipidus,^51^ quantifying vasopressin levels allows to determine the cause of the disease, which can be AVP receptor insensitivity or a decrease in hormone production. Notably, the preferred technique for detecting l-AVP in biomedical analyses has shifted to HPLC-MS/MS, sidelining traditional immunological techniques, partly due to its low molecular weight. This choice, however, requires essential preliminary steps for extracting and purifying the peptide of interest. The vasopressin nonapeptide possesses two cysteine residues at positions 1 and 6, forming a constrained peptide loop structure via a disulfide bridge. NMR analysis has revealed saddle and open conformations within the cyclic part of the molecule, while the noncyclic part demonstrates greater flexibility.^50^ The d-Arg-AVP (later named d-AVP) enantiomer represents a synthetic derivative of l-AVP, primarily employed for research purposes.^52^

Conversely, the C-terminally deaminated form of d-AVP, known as desmopressin, is used for therapeutic applications. Indeed, desmopressin selectively binds to a single receptor, V2, unlike the natural hormone l-AVP, limiting its physiological effects on water retention. Including d-Arg in the peptide chain makes it more resistant to proteolysis and enhances the molecule’s lifespan in the bloodstream.^53^

This work proves that a wild-type (WT) aerolysin nanopore can discriminate, at the single-molecule level, enantiomeric peptides, l-Arg8 or d-Arg8, contained in the native peptide chain. The l- and d-AVP can also be identified in a mixture. Interestingly, we can detect multiple AVP conformations, such as open and saddle states already observed by NMR. Furthermore, we show that after using a reducing agent several conformations observed in native conditions disappear, and the nanopore is still sensitive enough to identify both l- and d-forms in single and mixed experiments. By changing the relative ratio of each component in the mixture, we demonstrate the identification of each enantiomeric state according to its blockage level or volume. To identify each kind of event population and attribute them to different conformations, we used a PCA approach to first determine the best electrical parameters for their discrimination. We also developed a Monte Carlo prediction approach to identify each enantiomer conformation.

## Results and Discussion

### Electrical Enantiomer Characterization in Native Conditions

**Figure 1:**
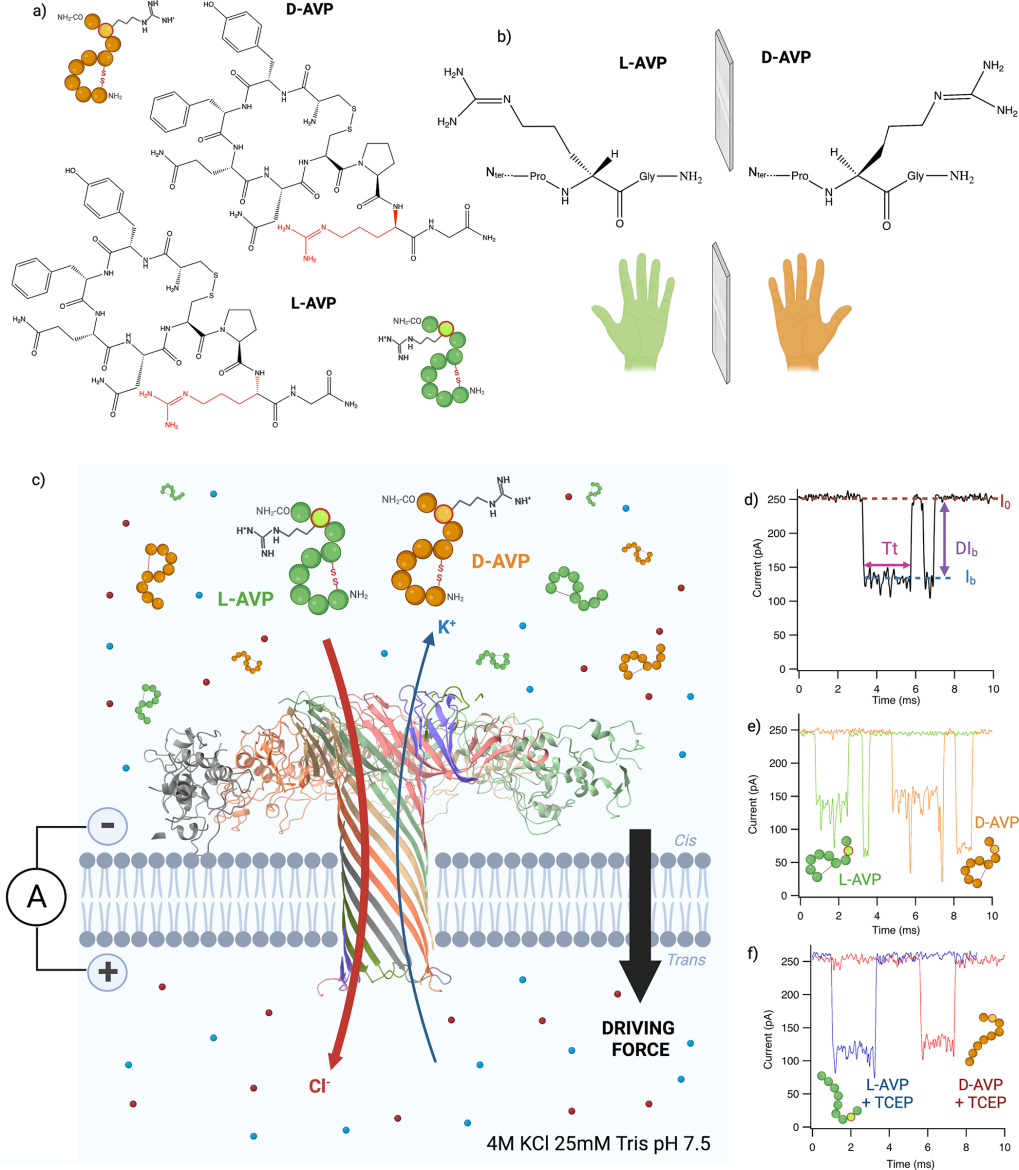
(a) Isomeric representation of l- and d-AVP (chemical structures created with ChemDraw). (b) Chemical representation of the chirality of vasopressin Arg8. (c) Representation of experimental conditions for the analysis of l-AVP (green) and d-AVP (orange) using a wild-type aerolysin nanopore (ribbon representation of PDB 5JZT using ChimeraX), inserted in a lipid bilayer in 4 M KCl and 25 mM Tris, pH 7.5. With an applied voltage, Cl^–^ and K^+^ ions go through the pore toward the oppositely charged electrodes, creating an ionic current (*I*_0_). (Created using BioRender.com.) (d) Example of an event showing the open pore current (*I*_0_) and blockade current (*I*_b_) resulting in a blockade level (DI_b_) over a dwell time (*T*_t_), characteristic of a peptide at 110 mV. (e) Example of the most representative types of events of l-AVP (green) and d-AVP (orange) at 110 mV. (f) Example of the most representative type of event of l-AVP (blue) and d-AVP (red) in the presence of 5 mM TCEP at 110 mV. (Image generated using Biorender.com.)

l- and d-AVP are two analogous peptides of 9 amino acids differing from a single chiral amino acid Arg8 (Figure 1a,b). Chirality can be represented as the non-superposable mirror image of an object. Therefore, the main difference between l- and d-AVP is the orientation of the lateral chain of Arg8 (Figure 1b). The two peptides form a disulfide bond between Cys1 and Cys6, creating a looped peptide structure (Figure 1a). As well as a disulfide bond, it has been found that l-AVP can possess one or two beta-turns stabilized by hydrogen bonds:^50^ the first one within the loop created by the disulfide bond creates the saddle conformation, and the other is formed across Pro7. l- and d-AVP structures are similar,^54^ so we can hypothesize that d-AVP can also form these two beta-turns. Discriminating l- and d-AVP with a WT aerolysin nanopore would enable us to show that we can detect a slight difference in conformation with a single chiral amino acid in structurally complex peptides. By reducing the disulfide bonds, we want to show if we can detect a conformational difference between the native and reduced states and determine the best conditions to discriminate between l- and d-AVP.

Using l- and d-AVP as model peptides, we want to show if with a wild-type aerolysin nanopore we can discriminate and identify a single amino acid enantiomer in both native and reducing conditions (Figure 1c). We performed nanopore experiments with l- and d-AVP, independently or in a mix, in the presence or absence of tris(2-carboxyethyl)phosphine (TCEP), a reducing agent. A WT aerolysin nanopore is inserted into a lipid bilayer separating two compartments (*cis* and *trans*) filled with an electrolyte solution (4 M KCl, 25 mM Tris, pH 7.5) (Figure 1c). A potential difference is applied by using two electrodes in each compartment. Ions in the electrolyte solutions flow through the pore, and we can measure an ionic current of 250 pA at 110 mV in our experimental conditions (*I*_0_). In the presence of an analyte at the pore entry, we observe current blockades (*I*_b_), characterized by a blockade level (DI_b_ = *I*_0_ – *I*_b_) and by a dwell time (*T*_t_) (Figure 1d). In native conditions, we observe two types of events for both l- and d-AVP: Type I with short dwell times of ∼440 and ∼390 μs, respectively, and a lower blockade current of ∼60 and ∼70 pA; and Type II with longer dwell times: for l-AVP ∼820 μs and d-AVP ∼1140 μs and a higher blockade current of ∼150 pA (Figure 1e) (Supporting Information S1, S2). In the presence of TCEP, a reducing agent, we observe only one type of event for each peptide characterized by long dwell times with a medium blockade current of ∼130 pA (d-AVP) and ∼120 pA (l-AVP) (Figure 1f). Interestingly, for these individual events, we can already observe a difference in the average blockade current for l- and d-AVP in either native or reducing conditions (Figure 1e and f, respectively).

**Figure 2:**
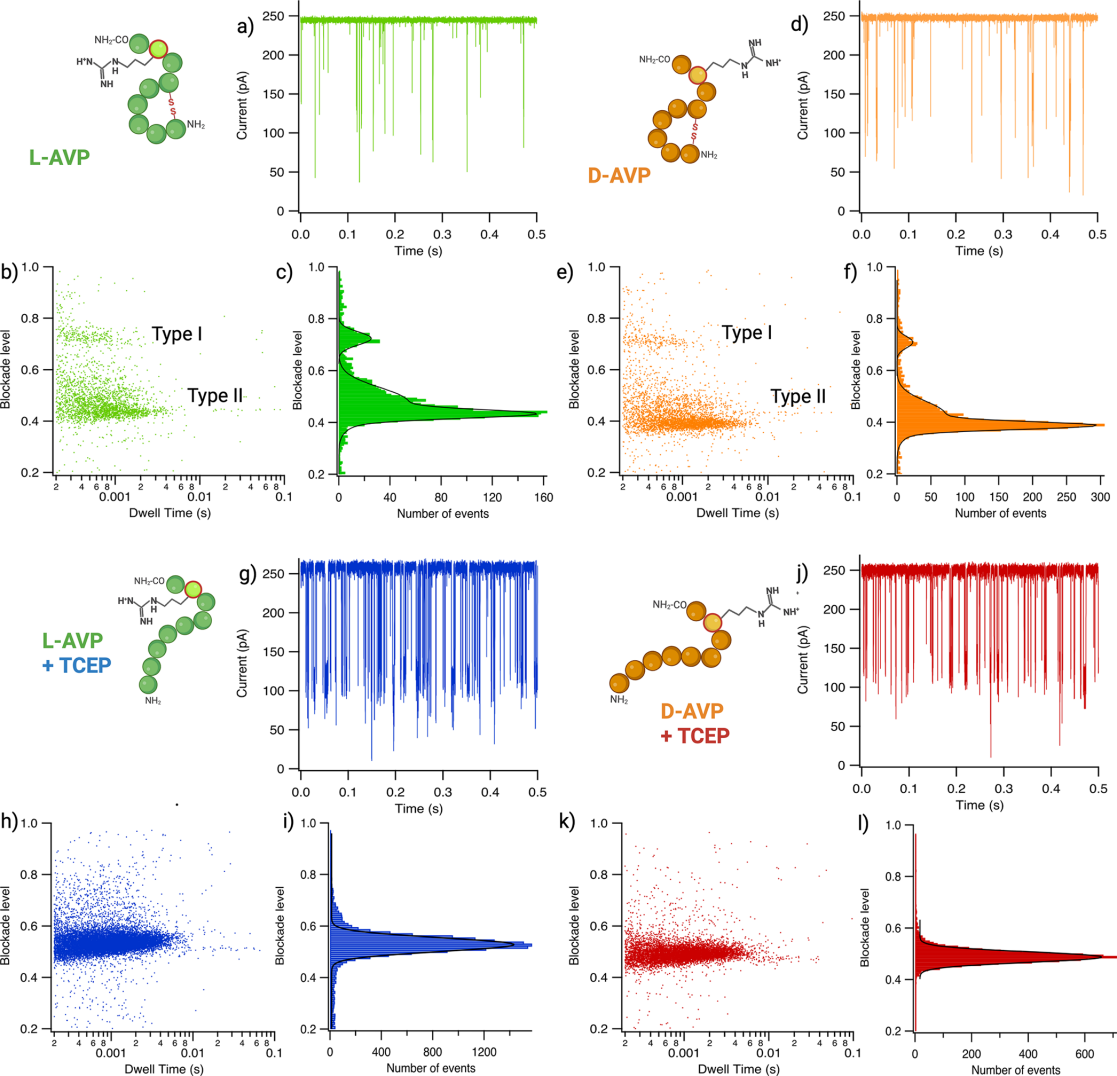
Experimental result of the independent analysis of 10 μM l- and d-AVP with or without 5 mM TCEP using an aerolysin nanopore in 4 M KCl 25 and mM Tris pH 7.5. (a, d, g, j) Example of current traces (filtered at 5 kHz) for each independent experimental condition at 110 mV: l-AVP (green), d-AVP (orange), l-AVP+TCEP (blue), and d-AVP+TCEP (red). Open pore currents, *I*_0_ = 243.82 ± 1.77 pA for l-AVP (a), *I*_0_ = 246.78 ± 2.04 pA for d-AVP (d), *I*_0_ = 259.22 ± 3.33 pA for l-AVP+TCEP (g), and *I*_0_ = 250.16 ± 3.46 pA for d-AVP+TCEP (j). (b, e, h, k) Scatter plot representing the normalized average blockade level (DI_b_) against the dwell time of each event longer than 200 μs between 0.2 and 1.0 in the blockade level representing the interacting population of each peptide in the absence and presence of TCEP. (Raw scatter plot can be found in Supporting Information S3 and S4.) (b, e) Scatter plot showing populations of events for l- and d-AVP in the absence of TCEP: Type I between 0.6 and 0.8 blockade level and Type II between 0.38 and 0.6 blockade level. (c, f, i, l) Histograms of normalized blockade levels as a function of the number of events fitted with a Gaussian function (black lines) to determine the most probable blockade level for Type I and a bi-Gaussian for Type II: l-AVP: Type I, 0.72 ± 0.01; Type IIa, 0.43 ± 0.01 for the population with the most events; Type IIb, 0.48 ± 0.01; and d-AVP: Type I, 0.71 ± 0.01; Type IIa, 0.39 ± 0.01; Type IIb, 0.43 ± 0.01. With TCEP average blockade levels fitted by a Gaussian for l-AVP + TCEP: 0.53 ± 0.01 and d-AVP + TCEP: 0.49 ± 0.01. Data shown are from a single recording, with fitted values being mean and standard deviation for three independent fits. *N*l-AVP = 2812 events; *N*d-AVP = 3961 events; *N*l-AVP+TCEP = 13383 events; *N*d-AVP+TCEP = 7644 events. The number of events was calculated by selecting the population with a dwell time superior to 200 μs, depending on their blockade level. (Image generated using Biorender.com.)

For experiments in native conditions, the characteristic parameters of dwell time and average blockade level for each event were extracted from the current traces and plotted as a bidimensional cloud (Figure 2a,b,d,e,g,h,j,k). Only the events of longer dwell time (>200 μs) are shown here, corresponding to the entrance of the peptides in the pore with a well-characterized blockade level as opposed to events with short dwell time (<200 μs) known as bumping events.^55−57^ We observed the two main types of events characterized by at least two defined current blockades, 0.43 ± 0.01 (Type IIa) and 0.72 ± 0.01 (Type I) for l-AVP, while 0.39 ± 0.01 (Type IIa) and 0.71 ± 0.01 (Type I) for d-AVP (Figure 2c,f) (Supporting Information S1). These results show that we can discriminate and identify l- and d-AVP in native conditions using the Type II events and their average current blockade. Unfortunately, the less frequent Type I events for l- and d-AVP cannot be discriminated. These events could be attributed to the entrance of the cycle formed by the disulfide bound. In fact, due to the volume of this cycle, we can suppose it is entropically unfavorable for entry into the pore. On the other hand, it could explain the highest blockade level observed. Furthermore, we observe a discrete subgroup in the Type II population that blocks the pore slightly more (Figure 2b,c,e,f). For l-AVP, we can measure a blockade level of 0.48 ± 0.01 (Type IIb) for this population and 0.43 ± 0.01 for d-AVP (Type IIb) (Supporting Information S1). Blockade levels depend on the volume of the chain interacting with or passing through the pore.^36,49,58,59^ Several studies showed that l-AVP could adopt multiple conformations. NMR studies^50,60^ showed that l-AVP can be either in a “saddle” or “open” conformation with a ratio of 70:30, respectively (Supporting Information S5). These two populations with similar observed blockade levels (Types IIa and IIb) could be due to this conformation change, with the most probable population observed being the saddle conformation, or Type IIa, and the less probable population being the open conformation, or Type IIb. These two conformations have different values for molecule volume.^50^ To better discriminate and identify each population, we will discuss this later in the paper by performing semisupervised classification to assign types of events to specific conformations.

### Electrical Enantiomer Characterization in Reducing Conditions

In experiments repeated in the presence of 5 mM TCEP, the bidimensional clouds for dwell time and blockade level display a single population of events with an average current blockade of 0.53 ± 0.01 for l-AVP and 0.49 ± 0.01 for d-AVP (Figure 2h,k; Supporting Information S1, S4). This single population in the presence of a reducing agent shows that we indeed have a change or loss of conformation. We confirmed that this single population is not due to TCEP affecting the detection of the peptides (Supporting Information S6). By comparing the previous experiments in native conditions, we can attribute events Type I and II to the disulfide bond. In the presence of the reducing agent, we observe that for both peptides the current blockade for Type II events increases, from 0.39 ± 0.01 for d-AVP to 0.49 ± 0.01 for d-AVP+TCEP and from 0.43 ± 0.01 for l-AVP to 0.53 ± 0.01 for l-AVP+TCEP (Figure 2i,j) (Supporting Information S1). The reduction of the disulfide bond releases the constraint on the peptide conformation, conferring more flexibility. To confirm these results, we analyzed and compared the event frequency in native and reducing conditions (Supporting Information S7). We can observe that event frequency at similar concentrations of peptide and applied voltage increased drastically after treatment with TCEP, from 19.1 ± 1.0 Hz to 153.3 ± 8.5 Hz for l-AVP at 110 mV in native and reducing conditions and 28.1 ± 0.9 and 107.2 ± 3.6 Hz for d-AVP, respectively. This result shows a reduced energy barrier for the entry of peptides treated with TCEP inside the nanopore. The reduction of the disulfide bond allows the peptide chain to adopt different conformations, which makes the peptide more flexible. This result is confirmed by an increase in the blockade level between the native and the reduced peptides (Figure 2c,f,i,l).

### Enantiomer Discrimination in a Mix

Since we observed a significant difference in blockade level between l- and d-AVP in native and reducing conditions, we performed equimolar mixes in both conditions to determine (1) if we can discriminate them in a mix and (2) what are the best experimental conditions for their discrimination (native or reducing conditions, applied voltage).

**Figure 3:**
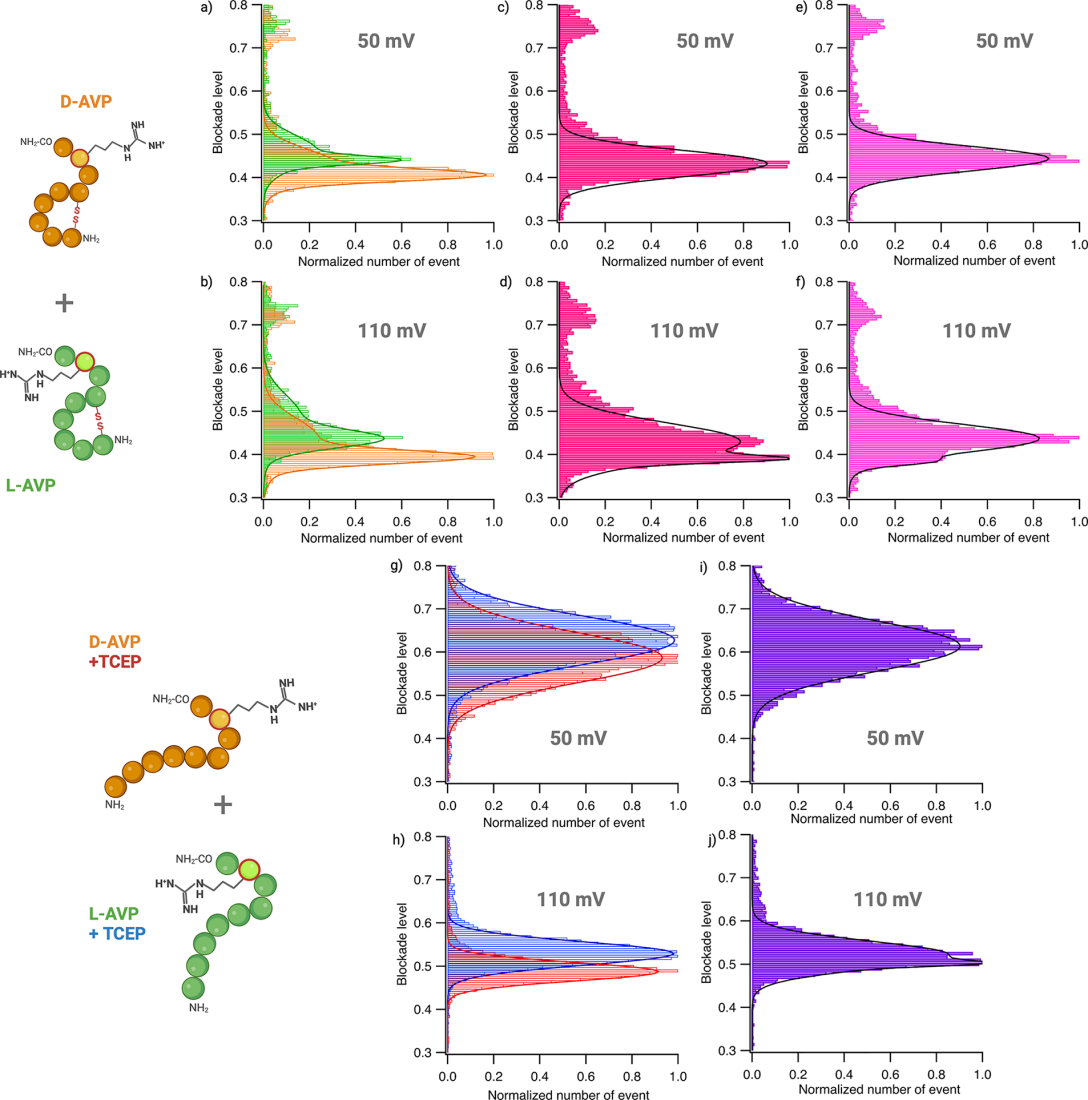
l- and d-AVP discrimination in a mix with and without TCEP using a wild-type aerolysin nanopore at 50 and 110 mV. Histograms of blockade levels from 0.3 to 0.8 as a function of the normalized total number of events fitted with a Gaussian or bi-Gaussian function (lines) to determine each population’s most probable blockade level. (Raw scatter plot can be found in Supporting Information S3 and S4.) (a, b) Superposition of l-AVP in green and d-AVP in orange (independent experiments at 10 μM). (c, d) 10 μM equimolar mix of l- and d-AVP (dark pink). (e, f) 7.5 μM l-AVP and 2.5 μM d-AVP (light pink). (g, i) Superposition of l-AVP with TCEP in blue and d-AVP with TCEP in red (independent experiments at 10 μM). (h, j) 5 μM equimolar mix of l- and d-AVP with 5 mM TCEP. (a, c, e, g, h) Experiments conducted at 50 mV. (b, d, f, i, j) Experiments conducted at 110 mV. (a) Independent blockade levels of Type IIa l-AVP = 0.43 ± 0.01 and Type IIb = 0.46 ± 0.01; Type IIa d-AVP = 0.40 ± 0.01 and Type IIb = 0.43 ± 0.01 at 50 mV. (b) Independent blockade levels of Type IIa l-AVP = 0.43 ± 0.01 and Type IIb 0.48 ± 0.01; Type IIa d-AVP = 0.39 ± 0.01 and Type IIb 0.43 ± 0.01 at 110 mV. (c) Blockade level measured: 0.43 ± 0.01 in an equimolar mix of l- and d-AVP at 50 mV. (d) Blockade levels measured: 0.39 ± 0.01 and 0.43 ± 0.01 representing Type IIa most probable mean blockade level of l- and d-AVP in an equimolar mix at 110 mV. (e) Blockade level measured: 0.44 ± 0.01 in a 75/25 concentration ratio mix of l- and d-AVP at 50 mV. (f) Blockade levels measured: 0.38 ± 0.01 and 0.44 ± 0.01 representing Type IIa most probable mean l- and d-AVP blockade levels in a 75/25 concentration ratio at 110 mV. (g) Independent blockade levels of l-AVP+TCEP = 0.62 ± 0.01 and d-AVP+TCEP = 0.58 ± 0.01 at 50 mV. (h) Independent blockade levels l-AVP+TCEP = 0.53 ± 0.01 and d-AVP+TCEP = 0.49 ± 0.01 at 110 mV. (i) Blockade level measured: 0.61 ± 0.01 in an equimolar mix of l- and d-AVP+TCEP at 50 mV. (j) Blockade level measured: 0.50 ± 0.01 and 0.52 ± 0.01 in an equimolar mix of l- and d-AVP+TCEP representing their respective blockade levels at 110 mV. Data shown are from a single recording with fitted values being mean and standard deviation for three independent fits. *N*l-AVP_50 mV = 2099 events; *N*d-AVP_50 mV = 1461 events; *N*l-AVP+TCEP_50 mV = 7849 events; *N*d-AVP+TCEP_50 mV = 5231 events; *N*l-AVP_110 mV = 2756 events; *N*d-AVP_110 mV = 3927 events; *N*l-AVP+TCEP_110 mV = 13227 events; *N*d-AVP+TCEP_110 mV = 7523 events; *N*l-d-AVP_eq_50 mV = 3832 events; *N*l-D_AVP_75/25_50 mV = 3226 events; *N*l-d-AVP+TCEP _eq_50 mV = 5460 events; *N*l-d-AVP_eq_110 mV = 4630 events; *N*l-D_AVP_75/25_110 mV = 5907 events; *N*l-d-AVP+TCEP _eq_110 mV = 4460 events. The number of events was calculated by selecting the population with a dwell time superior to 200 μs, depending on their blockade level. (Image generated using Biorender.com.)

We compared the superposition of the histograms of each independent experiment and the mixes (Figure 3a,b,g,h). In the absence of TCEP, we focused on the Type II events that allowed discrimination between the peptides. We varied the applied voltage (Supporting Information S8) and salt concentration (Supporting Information S9) to determine the best experimental conditions, with data at 50 and 110 mV in 4 M KCl displayed in Figure 3. At 50 mV, the blockade level histograms of l- and d-AVP in native or reducing conditions overlap (Figure 3a,c,e,g,i). Indeed, in a mix, we could not discriminate between each peptide population at this voltage. By increasing the voltage to 110 mV, the blockade levels for each peptide were better resolved (Figure 3b,d,f,h,j).

We measured the most probable mean blockade level for both mixes (Supporting Information S10). In native conditions, blockade levels of 0.39 ± 0.01 and 0.43 ± 0.01 were measured, while with TCEP they were found to be 0.49 ± 0.01 and 0.53 ± 0.01 (Figure 3d,j) (Supporting Information S11), confirming that we can discriminate l- and d-AVP in an equimolar mix in both conditions. To verify that each population observed is attributed to l- or d-AVP, we changed the concentration ratio to 75% l-AVP and 25% d-AVP (Figure 3e,f). We observed a decrease in the number of events for the population attributed to d-AVP compared to l-AVP. To further confirm this, we changed the ratio to 25% l-AVP and 75% d-AVP and showed the same ability to discriminate between the peptides (Supporting Information S12). Here, we demonstrated that we could discriminate and identify l- and d-AVP, two native or reduced peptides differing only by one chiral amino acid in a mix using a WT aerolysin nanopore at 110 mV.

### Identification by Principal Component Analysis and Monte Carlo Approaches

**Figure 4:**
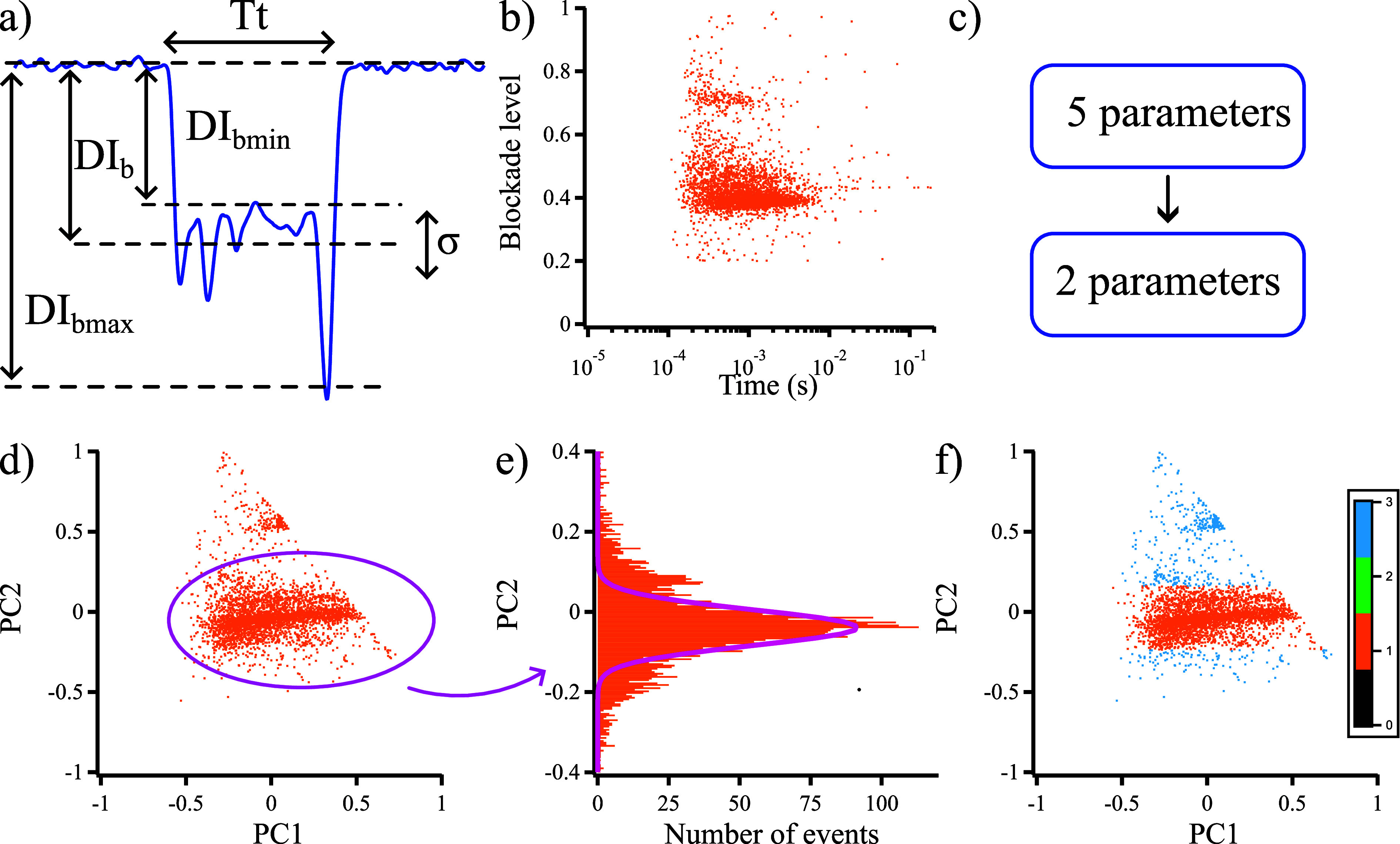
Principal component analysis (PCA). (a) Parameters used to characterize each blockade minimum, average, maximum blockades (DI_bmin_, DI_b_, DI_bmax_), standard deviation (σ), and duration (*T*_t_) of each blockade. (b) Scatter plot of the average blockade of each d-AVP blockade according to its duration. (c) PCA strategy to decrease the parameter numbers. (d) Second principal component (PC2) according to the first one (PC1). (e) PC2 histogram fitted by a Gaussian distribution (μ_D_ = 0.0474, σ_D_ = 0.0348, describing the most probable value and the standard deviation of the distribution, respectively). (f) Selection of the relevant blockades in the range [μ_D_ ± σ_D_] in 10 μM d-AVP using an aerolysin nanopore in 4 M KCl and 25 mM Tris, pH 7.5, *V* = 110 mV.

In order to accurately identify each enantiomeric peptide and their different conformations, we performed a principal component analysis (PCA). For the experimental data analysis, we considered just the blockade level DI_b_ to characterize each blockade. We can go further by better characterizing the shape of the blockades using additional parameters.^41−43^ Then, we can consider four other parameters defined in Figure 4a: maximum and minimum blockades (DI_bmax_, DI_bmin_, respectively), duration (*T*_t_), and standard deviation (σ). We removed the blockades characterized by a standard deviation smaller than 1 pA, which were due to bumping, or by a blockade level smaller than 0.2 (see above). These data are plotted in Figure 4b from the results obtained with 10 μM d-AVP and show two main clusters: a small one characterized by a high blockade level of around 0.7 and a large one around 0.4.

If we want to take the five parameters together into account, it is necessary to perform a PCA to reduce the number of relevant parameters. Then, we consider the two most relevant parameters: the first and second principal components (PC1 and PC2, respectively; Figure 4e). We observe two clusters similar to those in Figure 4b: a small cluster and a large one. We are particularly interested in the second principal component (PC2) and its Gaussian distribution for the larger population, which is similar to the blockade levels (Figure 2c,f). The two peptides have very similar structures, so the corresponding distributions are very close. These two distributions overlap, making classical clustering analyses with well-separated domains impossible (Supporting Information S13).

To overcome this difficulty, we considered the results obtained from experiments with individual peptides. As a first approximation, each distribution is fitted by a Gaussian centered at μ_D_ (respectively μ_L_) with a standard deviation of σ_D_ (respectively σ_L_) for the d-AVP (respectively l-AVP) peptides. Blockades belonging to the domain [μ-3·σ, μ+3·σ] are attributed to the corresponding peptide (d-AVP or l-AVP), allowing the classification of 99% of blockades (Figure 4e,f and Supporting Information S13). Fitting the histogram of the main PC2 population for the d-AVP like this determines μ_D_ = −0.039 and σ_D_ = 0.043 (Figure 4e). The classification is performed by taking into account all of the blockades in the range [μ_D_-3·σ_D_, μ_D_+3·σ_D_]. These data are colored in orange. The remaining data are not labeled and are in cyan (Figure 4f).

We followed the same approach with the l-AVP experimental data using the correlation matrix previously calculated from d-AVP data. We can observe a similar behavior: a PC2 distribution fitted by a Gaussian function as before, where μ_L_ = 0.047 and σ_L_ = 0.035 are the fitted values for l-AVP. All the blockades in the range [μ_L_ – 3*σ_L_, μ_L_ + 3*σ_L_] are attributed to l-AVP peptides (in green). The other blockades are unassigned (in cyan) (Supporting Information S13).

**Figure 5:**
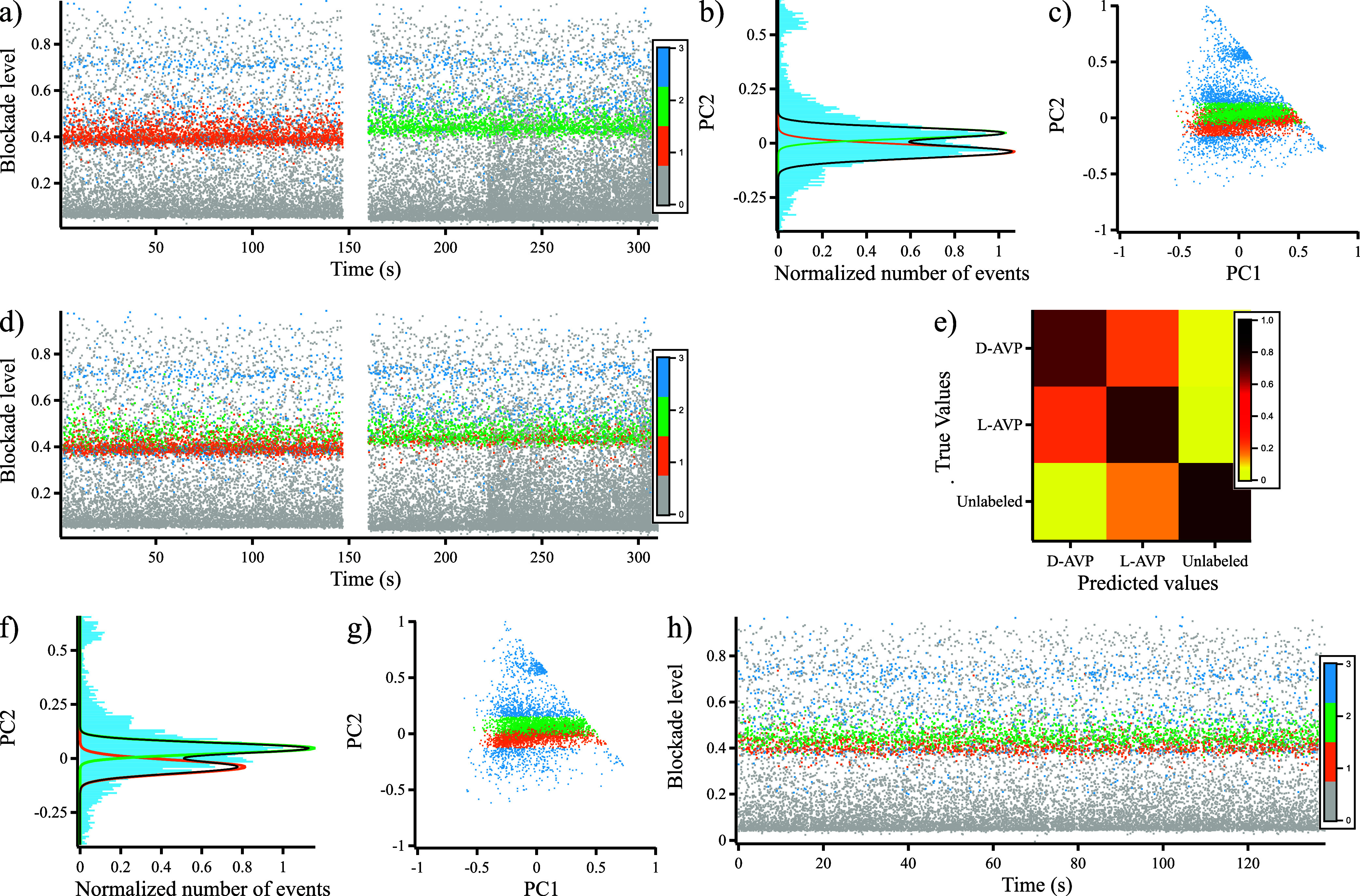
Prediction of d- and l-AVP mixtures. (a) Combined data from blockade traces obtained for d- (orange) or l-AVP (green), respectively. The gray events correspond to the ones with σ < 1 pA (10 μM d- or l-AVP using an aerolysin nanopore in 4 M KCl and 25 mM Tris, pH 7.5, *V* = 110 mV). (b) Distribution of the second principal component (PC2), fitted by bi-Gaussian (gray) or Gaussian (orange or green) curves. (c) Scatter plot of the two principal components. The area colored in orange or green corresponds to the bands centered in μ_D_ (μ_L_) and with a width of ±3 σ_D_ (σ_L_). It is labeled according to the nature of each isomer. (d) Evaluation of Monte Carlo prediction. Predicted blockade trace according to four types of labeling obtained from the data used in (a). (e) Confusion matrix calculated from the Monte Carlo prediction. (f) Equimolar mixture of 10 μM d- and l-AVP distribution of the second principal component (PC2), fitted by bi-Gaussian (gray) or Gaussian (orange or green) curves. Δ*V* = 110 mV. (g) Scatter plot of the two principal components. The area colored in orange or green is calculated according to a Monte Carlo algorithm. (h) Blockade trace according to four types of labeling: σ < 1 (gray), d-AVP (orange), l-AVP (green), and not attributed (cyan).

We used these criteria to discriminate between d-AVP and l-AVP in a mixture. First, we combined data from d-AVP and l-AVP current traces to define the training data (Figure 5a). These classifications led to labeling the previous data combined (Figure 5a). The PCA is computed using the correlation matrix previously calculated from d-AVP data. The PC2 distribution shows the two Gaussian peaks previously observed for each peptide (Figure 5b). As both distributions are overlaid, the logistic regression approach to define clusters is irrelevant (Supporting Information S14). We follow a Monte Carlo approach to discriminate the two peptides and label them (Figure 5c), followed by a comparison of our prediction with the original labeling of d-AVP and l-AVP by calculating a confusion matrix (Figure 5e). The success rates are 71% for d-AVP and 75% for l-AVP recognition. Nevertheless, the false-positive rates are similar for both peptides (around 22%) due to the overlap in the two distributions.

We applied this approach to data collected from an equimolar mixture (10 μM d-AVP and l-AVP). The PC2 distribution (Figure 5f) is similar to the one observed for the combined data from d-AVP and l-AVP in Figure 5b. We used the same criteria as used previously to label the blockades: 1 = d-AVP (orange), 2 = l-AVP (green), and 3 = unlabeled data (cyan). Figure 5g shows the scatter plot of the two first-principal components according to this labeling, which leads to the blockade trace represented in Figure 5h.

We applied this method to nonequimolar mixtures (2.5 μM l-AVP/7.5 μM d-AVP and 7.5 μM l-AVP/2.5 μM d-AVP), leading to the ratio calculation of the number of l-AVP blockades divided by that of d-AVP blockades (Supporting Information S15). The correlation between this ratio and the relative composition of the mixtures (1:3, 1:1, and 1:3 l-AVP:d-AVP, respectively) shows the accuracy of our approach.

Using five parameters provides a more comprehensive view of the blockade phenomena and allows the determination of the most relevant parameters. The correlation matrix calculated from the d-AVP data was systematically used for each analysis. The scatter plots representing the first two principal components show a structure with several domains (a large and a small) comparable to classic representations of blockade levels versus durations, whether for d-AVP or l-AVP peptides.

In the case of data collected from a mixture, we followed a Monte Carlo approach to assign the blockades to one peptide or the other (Figure 5f). Our approach was validated using the combined data sets from experiments performed with the l-AVP and d-AVP peptides separately. The corresponding confusion matrix shows a success rate of 73%, which is explained by the overlapping between the two distributions. This result is interesting and demonstrates the relevance of this approach in the statistical analysis of peptides by nanopores.

Furthermore, these blockade level distributions (Figure 2c,f) highlight a shoulder attributed to the open conformation.^50^ The PC2 distributions clearly show two symmetrical shoulders for d-AVP (Figure 4e), whereas there is only one for l-AVP (Supporting Information S13). Then, we could also discriminate the two peptides from the PC2 shape distribution. Principal component analyses are very promising for further analysis of the different conformations of peptides at the single-molecule level.

## Conclusion

The world’s population is increasing, and people worldwide are living longer. The degradation of the environment also impacts health. In this context, we expect an increase in chronic diseases, cancers, and neurodegenerative diseases.^40^ To answer this health challenge, we need a powerful, sensitive, and specific tool to perform early disease detection. Many molecules, including established peptide and protein clinical biomarkers and yet-to-be-identified biomarkers, remain underutilized or unexploited for clinical applications. This is due to the absence of techniques capable of discerning different conformations, post-translational modifications, and d/l amino acids. This study paves the way for the ability to identify, characterize, and quantify free or contained d-amino acids in peptides or proteins, which will be crucial for the early detection of human diseases. From a chemical and pharmaceutical point of view, this technique would also allow the scientific community to discriminate isomers^61^ or follow the conversion of molecule chirality^62^ and explain nonenzymatic racemization pathways.^63^ While this study focuses on a specific peptide model, there is no doubt that this technique can be extended to other enantiomer peptides (lanthipeptide and Aβ peptides), as shown in two recent publications using different protein nanopores such as OmpF,^48^ CytK, and FraC.^49^

We showed that we can detect and discriminate biologically relevant peptides differing by a single amino-acid enantiomer, l- or d-Arg. The peptides can be identified individually or as a mixture. We detected two different peptide conformations, saddle (Type IIa) and open (Type IIb), which NMR already described. Due to their unique conformation, we could identify each peptide using two electrical parameters: dwell time and current blockade. Using a reducing agent, we did not detect any more conformational variants observed under native conditions. On the other hand, we can still identify l- and d-AVP that adopt different conformations. We also used a PCA approach to confirm our experimental data analysis and define the best electrical parameters to discriminate each population of events. This method is up-and-coming and will make it possible to study the conformations of biomarkers in more detail at the single-molecule level.

## Methods

### Peptides

Vasopressin peptides, l-Arg vasopressin (l-AVP), Nter-C_1_-Y_2_-F_3_-Q_4_-N_5_-C_6_-P_7_-_(L)_R_8_-G_9_-CO-NH_2_, and d-Arg vasopressin (d-AVP), Nter-C_1_-Y_2_-F_3_-Q_4_-N_5_-C_6_-P_7_-_(D)_R_8_-G_9_-CO-NH_2_, were synthesized and purified by HPLC (Proteogenics, Schiltigheim, France) with a disulfide bond between C_1_–C_6_, dissolved in 25 mM Tris pH 7.5 to either 1 or 10 mM, and aliquoted and stored at −20 °C until use. The purity analysis of the l-AVP (Supporting Information S16) and d-AVP (Supporting Information S17) is checked by mass spectrometry and liquid chromatography.

### Chemicals

Tris(2-carboxyethyl)phosphine (ref: T2556), hydrochloride (TCEP) was synthesized by Invitrogen (Thermo Fisher Scientific) and dissolved in 100 mM Tris pH 7.5 to 1 M and aliquoted and stored at −20 °C until use.

### Protein Production

Proaerolysin was produced by Dreampore SAS (Cergy, France) as described previously.^21^ Briefly, C-terminally His-tagged protein was expressed into the periplasm of BL21 Ros 2 cells (MilliporeSigma), harvested by osmotic shock, before further purification by nickel affinity and buffer exchange chromatography (Cytiva, Malborough MA, USA) in standard buffers containing 350 mM NaCl. Purified protein was stored at 4 °C until further use, when it was activated using trypsin-bound beads (Thermo Scientific, Waltham, MA, USA). It was then used in nanopore experiments at a 0.5–1 nM final concentration of aerolysin monomers.

### Nanopore Experiments

Nanopore experiments were performed using a vertical planar lipid bilayer setup (Warner Instruments, Hamden CT, USA) with a 150 mM aperture, as described previously.^57^ A 10 mg/mL stock of diphytanoyl-phosphatidyl-choline (DphPC, Avanti Polar Lipids, Alabaster, AL, USA), dissolved in decane, was used to create a planar lipid bilayer separating compartments containing 1 mL of electrolyte solution, 4 M KCl, and 25 mM Tris, pH 7.4. Once a single aerolysin pore was inserted, peptide analytes (individually or in a mix) were introduced at different concentrations before a 110 or 50 mV voltage differential was applied through Ag/AgCl electrodes.

### Data Acquisition and Analysis

Electrical measurements were performed by using an Axopatch 200B and a Digidata 1440 digitizer. Data were recorded at 250 kHz or 4 μs sampling time and filtered at 5 kHz using Clampex software (Axon Instruments, Union City, CA, USA). Three-minute recordings taken at 50 or 110 mV were cleaned and analyzed with Igor Pro (Wavemetrics, Portland, OR, USA), using in-house algorithms to detect events and extract their characteristic parameters (current baseline level *I*_0_, values for the average (*I*_b_), maximum (*I*_bmax_), and minimum (*I*_bmin_) current blockade level within the event, event noise or standard deviation of the current values within the event (*I*_bs_), dwell time, and blockade time location in the data set. The average open pore current (*I*_0_) and standard deviation (σ) for each recording were determined statistically,^64^ and a threshold of *I*_0_ – 7σ was used to define events for the target peptides in the characterization experiments (Figures 2, 3). Extracted parameters from multiple recordings were concatenated for low-frequency events to provide sufficient information for robust statistics. To remove bias introduced by short bumping events with a high blockade fraction, events with dwell times equal to or greater than 200 μs were used for further analysis. This is a reasonable approach given the overall distribution of average blockade fraction vs dwell time data (Supporting Information S2, S3), relative length of event dwell times, and the use of a 5 kHz filter. Histograms of the average blockade fraction for each event (DI_b_/*I*_0_, where DI_b_ = *I*_0_ – *I*_b_; binned at 0.005) were fit with Gaussian or bi-Gaussian functions to determine the average blockade fraction for each population of events. Semilog histograms of the number of events against dwell time, with 30 bins per decade, were fit with an exponential function to determine the most probable dwell time (Supporting Information S2). Semilog distributions of interevent time, binned at 2 ms for global frequency and 4 ms for population frequency, were fit with single-exponential functions to determine the event frequency (Supporting Information S7). Each experiment contained at least 1700 events of more than 200 μs in dwell time. Depending on their blockade level distribution, populations were selected to determine each mean dwell time value. For Type I (l- and d-AVP), the chosen points were found between 0.7 and 0.8 blockade level, and for Type II, between 0.35 and 0.55. In the presence of TCEP, points were selected between 0.4 and 0.8. The average and standard deviation determined from 3 independent fits were used for each fit to account for fit robustness.

### Semisupervised Clustering Using Principal Component Analysis and Logistic Regression Classification

The five parameters (*T*_t_, DI_bmin_, DI_bmax_, DI_b_, and *I*_bs_) were determined (Figure 5b) using DI_bmin_ = *I*_0_ – *I*_bmax_, DI_bmax_ = *I*_0_ – *I*_bmin_, and DI_b_ = *I*_0_ – *I*_b,_ respectively. PCA was performed using the PCA module of the scikit-learn library in Python.^65,66^ Briefly, the covariance matrix between these previously normalized five parameters was calculated to determine this matrix’s eigenvalues and vectors. Each parameter is projected onto each eigenvector, with only the eigenvectors characterized by the most significant eigenvalues (PC1 and PC2) used for further analysis. The calculations with l-AVP or mixtures were performed using the correlation matrix already calculated with d-AVP. The logistic regression classification^67^ was performed using the corresponding module in the scikit-learn Python library. The training is performed with 20% of the data set composed by concatenating data from d-AVP and l-AVP experiments (without or with TCEP). We can also follow a Monte Carlo type approach. We first used the histograms of PC2 obtained with each d-AVP or l-AVP peptide. These histograms are fitted by distinct Gaussian distributions centered in μ with a standard sigma deviation. We used these two Gaussian distributions to classify the blockages of a mixture according to a Monte Carlo approach. This method is evaluated using data obtained after the concatenation of data obtained previously with d-AVP and l-AVP peptides. We only take into account the blockages included in the band [μ-3·σ, μ+3·σ] of each Gaussian distribution. The predictions are compared to the true initial values to evaluate this method and calculate the corresponding confusion matrix using the corresponding sci-kit-learn library in Python.

## Supporting Information Available

Additional experimental details and results as well as tables regrouping population characteristics in each condition (PDF)

## References

[ref1] Abdulbagi M.; Wang L.; Siddig O.; Di B.; Li B. d-Amino Acids and d-Amino Acid-Containing Peptides: Potential Disease Biomarkers and Therapeutic Targets?. Biomolecules 2021, 11 (11), 1716 10.3390/biom11111716. 34827714 PMC8615943

[ref2] Mothet J. P. Physiological Relevance of Endogenous Free d-Serine in the Mammalian Brain: Are Scientists on a Royal Road for the Treatment of Glutamatergic-Related Brain Disorders?. Pathol. Biol. 2001, 49 (8), 655–659. 10.1016/S0369-8114(01)00227-9. 11692754

[ref3] Ploux E.; Freret T.; Billard J.-M. d-Serine in Physiological and Pathological Brain Aging. Biochim. Biophys. Acta (BBA) - Proteins Proteom. 2021, 1869 (1), 140542 10.1016/j.bbapap.2020.140542. 32950692

[ref4] Li Y.; Han H.; Yin J.; Li T.; Yin Y. Role of d-Aspartate on Biosynthesis, Racemization, and Potential Functions: A Mini-Review. Anim. Nutr. 2018, 4 (3), 311–315. 10.1016/j.aninu.2018.04.003. 30175260 PMC6116324

[ref5] Orzylowski M.; Fujiwara E.; Mousseau D. D.; Baker G. B. An Overview of the Involvement of d-Serine in Cognitive Impairment in Normal Aging and Dementia. Front. Psychiatry 2021, 12, 754032 10.3389/fpsyt.2021.754032. 34707525 PMC8542907

[ref6] Ota N.; Shi T.; Sweedler J. V. d-Aspartate Acts as a Signaling Molecule in Nervous and Neuroendocrine Systems. Amino Acids 2012, 43 (5), 1873–1886. 10.1007/s00726-012-1364-1. 22872108 PMC3555687

[ref7] Fujii N.; Tajima S.; Tanaka N.; Fujimoto N.; Takata T.; Shimo-Oka T. The Presence of d-β-Aspartic Acid-Containing Peptides in Elastic Fibers of Sun-Damaged Skin: A Potent Marker for Ultraviolet-Induced Skin Aging. Biochem. Biophys. Res. Commun. 2002, 294 (5), 1047–1051. 10.1016/S0006-291X(02)00597-1. 12074583

[ref8] Fujii N.; Satoh K.; Harada K.; Ishibashi Y. Simultaneous Stereoinversion and Isomerization at Specific Aspartic Acid Residues in ΑA-Crystallin from Human Lens. J. Biochem. 1994, 116 (3), 663–669. 10.1093/oxfordjournals.jbchem.a124577. 7852288

[ref9] Roher A. E.; Lowenson J. D.; Clarke S.; Wolkow C.; Wang R.; Cotter R. J.; Reardon I. M.; Zürcher-Neely H. A.; Heinrikson R. L.; Ball M. J. Structural Alterations in the Peptide Backbone of Beta-Amyloid Core Protein May Account for Its Deposition and Stability in Alzheimer’s Disease. J. Biol. Chem. 1993, 268 (5), 3072–3083. 10.1016/S0021-9258(18)53661-9. 8428986

[ref10] Sheykhkarimli D.; Choo K.-L.; Owen M.; Fiser B.; Jójárt B.; Csizmadia I. G.; Viskolcz B. Molecular Ageing: Free Radical Initiated Epimerization of Thymopentin - A Case Study. J. Chem. Phys. 2014, 140 (20), 205102 10.1063/1.4871684. 24880333

[ref11] Kuge K.; Kitamura K.; Nakaoji K.; Hamada K.; Fujii N.; Saito T.; Fujii N. Oxidative Stress Induces the Formation of d-Aspartyl Residues in the Elastin Mimic Peptides. Chem. Biodivers. 2010, 7 (6), 1408–1412. 10.1002/cbdv.200900348. 20564559

[ref12] Yan J.; Yao Y.; Yan S.; Gao R.; Lu W.; He W. Chiral Protein Supraparticles for Tumor Suppression and Synergistic Immunotherapy: An Enabling Strategy for Bioactive Supramolecular Chirality Construction. Nano Lett. 2020, 20 (8), 5844–5852. 10.1021/acs.nanolett.0c01757. 32589431

[ref13] Fujii N.; Takata T.; Fujii N.; Aki K.; Sakaue H. d-Amino Acids in Protein: The Mirror of Life as a Molecular Index of Aging. Biochim. Biophys. Acta (BBA) - Proteins Proteom. 2018, 1866 (7), 840–847. 10.1016/j.bbapap.2018.03.001. 29530565

[ref14] Lander A. J.; Jin Y.; Luk L. Y. P. d-Peptide and d-Protein Technology: Recent Advances, Challenges, and Opportunities**. ChemBioChem. 2023, 24 (4), e202200537 10.1002/cbic.202200537. 36278392 PMC10805118

[ref15] Oukhaled G.; Mathe J.; Biance A. L.; Bacri L.; Betton J.-M.; Lairez D.; Pelta J.; Auvray L. Unfolding of Proteins and Long Transient Conformations Detected by Single Nanopore Recording. Phys. Rev. Lett. 2007, 98 (15), 158101–158101. 10.1103/PhysRevLett.98.158101. 17501386

[ref16] Merstorf C.; Cressiot B.; Pastoriza-Gallego M.; Oukhaled A.; Betton J.-M.; Auvray L.; Pelta J. Wild Type, Mutant Protein Unfolding and Phase Transition Detected by Single-Nanopore Recording. ACS Chem. Biol. 2012, 7 (4), 652–658. 10.1021/cb2004737. 22260417

[ref17] Galenkamp N. S.; Biesemans A.; Maglia G. Directional Conformer Exchange in Dihydrofolate Reductase Revealed by Single-Molecule Nanopore Recordings. Nat. Chem. 2020, 167, 378–8. 10.1038/s41557-020-0437-0. 32251371

[ref18] Boersma A. J. A.; Bayley H. H. Continuous Stochastic Detection of Amino Acid Enantiomers with a Protein Nanopore. Angew. Chem., Int. Ed. Engl. 2012, 51 (38), 9606–9609. 10.1002/anie.201205687. 22930401

[ref19] He L.; Tessier D. R.; Briggs K.; Tsangaris M.; Charron M.; McConnell E. M.; Lomovtsev D.; Tabard-Cossa V. Digital Immunoassay for Biomarker Concentration Quantification Using Solid-State Nanopores. Nat. Commun. 2021, 12 (1), 5348 10.1038/s41467-021-25566-8. 34504071 PMC8429538

[ref20] Chuah K.; Wu Y.; Vivekchand S. R. C.; Gaus K.; Reece P. J.; Micolich A. P.; Gooding J. J. Nanopore Blockade Sensors for Ultrasensitive Detection of Proteins in Complex Biological Samples. Nat. Commun. 2019, 10 (1), 2109 10.1038/s41467-019-10147-7. 31068594 PMC6506515

[ref21] Stierlen A.; Greive S. J.; Bacri L.; Manivet P.; Cressiot B.; Pelta J. Nanopore Discrimination of Coagulation Biomarker Derivatives and Characterization of a Post-Translational Modification. Acs Central Sci. 2023, 9 (2), 228–238. 10.1021/acscentsci.2c01256. PMC9951287 36844502

[ref22] Lucas F. L. R.; Versloot R. C. A.; Yakovlieva L.; Walvoort M. T. C.; Maglia G. Protein Identification by Nanopore Peptide Profiling. Nat. Commun. 2021, 12 (1), 5795 10.1038/s41467-021-26046-9. 34608150 PMC8490355

[ref23] Huang G.; Voorspoels A.; Versloot R. C. A.; Heide N. J.; Carlon E.; Willems K.; Maglia G. PlyAB Nanopores Detect Single Amino Acid Differences in Folded Haemoglobin from Blood**. Angewandte Chemie Int. Ed 2022, 61 (34), e202206227 10.1002/anie.202206227. PMC9541544 35759385

[ref24] Ahmad M.; Ha J.-H.; Mayse L. A.; Presti M. F.; Wolfe A. J.; Moody K. J.; Loh S. N.; Movileanu L. A Generalizable Nanopore Sensor for Highly Specific Protein Detection at Single-Molecule Precision. Nat. Commun. 2023, 14 (1), 1374 10.1038/s41467-023-36944-9. 36941245 PMC10027671

[ref25] Huang G.; Willems K.; Soskine M.; Wloka C.; Maglia G. Electro-Osmotic Capture and Ionic Discrimination of Peptide and Protein Biomarkers with FraC Nanopores. Nat. Commun. 2017, 8 (1), 935 10.1038/s41467-017-01006-4. 29038539 PMC5715100

[ref26] Galenkamp N. S.; Soskine M.; Hermans J.; Wloka C.; Maglia G. Direct Electrical Quantification of Glucose and Asparagine from Bodily Fluids Using Nanopores. Nat. Commun. 2018, 9 (1), 4085 10.1038/s41467-018-06534-1. 30291230 PMC6173770

[ref27] Jiang J.; Li M.-Y.; Wu X.-Y.; Ying Y.-L.; Han H.-X.; Long Y.-T. Protein Nanopore Reveals the Renin-Angiotensin System Crosstalk with Single-Amino-Acid Resolution. Nat. Chem. 2023, 1–9. 10.1038/s41557-023-01139-8. 36805037

[ref28] Greive S. J.; Bacri L.; Cressiot B.; Pelta J. Identification of Conformational Variants for Bradykinin Biomarker Peptides from a Biofluid Using a Nanopore and Machine Learning. ACS Nano 2024, 18 (1), 539–550. 10.1021/acsnano.3c08433. 38134312

[ref29] Zhang M.; Tang C.; Wang Z.; Chen S.; Zhang D.; Li K.; Sun K.; Zhao C.; Wang Y.; Xu M. J.; et al. Real-Time Detection of 20 Amino Acids and Discrimination of Pathologically Relevant Peptides with Functionalized Nanopore. Nat. Methods 2024, 1–10. 10.1038/s41592-024-02208-7. 38443507 PMC11009107

[ref30] Restrepo-Pérez L.; Wong C. H.; Maglia G.; Dekker C.; Joo C. Label-Free Detection of Post-Translational Modifications with a Nanopore. Nano Lett. 2019, 19 (11), 7957–7964. 10.1021/acs.nanolett.9b03134. 31602979 PMC6856961

[ref31] Versloot R. C. A.; Lucas F. L. R.; Yakovlieva L.; Tadema M. J.; Zhang Y.; Wood T. M.; Martin N. I.; Marrink S. J.; Walvoort M. T. C.; Maglia G. Quantification of Protein Glycosylation Using Nanopores. Nano Lett. 2022, 22 (13), 5357–5364. 10.1021/acs.nanolett.2c01338. 35766994 PMC9284675

[ref32] Restrepo-Pérez L.; Huang G.; Bohländer P. R.; Worp N.; Eelkema R.; Maglia G.; Joo C.; Dekker C. Resolving Chemical Modifications to a Single Amino Acid within a Peptide Using a Biological Nanopore. ACS Nano 2019, 13 (12), 13668–13676. 10.1021/acsnano.9b05156. 31536327 PMC6933820

[ref33] Huo M.; Hu Z.; Ying Y.; Long Y. Enhanced Identification of Tau Acetylation and Phosphorylation with an Engineered Aerolysin Nanopore. Proteomics 2022, 22 (5–6), 2100041 10.1002/pmic.202100041. 34545670

[ref34] Meng F.-N.; Ying Y.-L.; Yang J.; Long Y.-T. A Wild-Type Nanopore Sensor for Protein Kinase Activity. Anal. Chem. 2019, 91 (15), 9910–9915. 10.1021/acs.analchem.9b01570. 31241901

[ref35] Ying Y.-L.; Yang J.; Meng F.-N.; Li S.; Li M.-Y.; Long Y.-T. A Nanopore Phosphorylation Sensor for Single Oligonucleotides and Peptides. Research (Wash D C) 2019, 2019 (8), 1050735–1050738. 10.34133/2019/1050735. 31912023 PMC6944226

[ref36] Ouldali H.; Sarthak K.; Ensslen T.; Piguet F.; Manivet P.; Pelta J.; Behrends J. C.; Aksimentiev A.; Oukhaled A. Electrical Recognition of the Twenty Proteinogenic Amino Acids Using an Aerolysin Nanopore. Nat. Biotechnol. 2019, 31, 247–6. 10.1038/s41587-019-0345-2. PMC7008938 31844293

[ref37] Wang K.; Zhang S.; Zhou X.; Yang X.; Li X.; Wang Y.; Fan P.; Xiao Y.; Sun W.; Zhang P.; et al. Unambiguous Discrimination of All 20 Proteinogenic Amino Acids and Their Modifications by Nanopore. Nat. Methods 2023, 1–10. 10.1038/s41592-023-02021-8. 37749214

[ref38] Cressiot B.; Bacri L.; Pelta J. The Promise of Nanopore Technology: Advances in the Discrimination of Protein Sequences and Chemical Modifications. Small Methods 2020, 4 (11), 2000090 10.1002/smtd.202000090.

[ref39] Ying Y.-L.; Hu Z.-L.; Zhang S.; Qing Y.; Fragasso A.; Maglia G.; Meller A.; Bayley H.; Dekker C.; Long Y.-T. Nanopore-Based Technologies beyond DNA Sequencing. Nat. Nanotechnol. 2022, 17 (11), 1136–1146. 10.1038/s41565-022-01193-2. 36163504

[ref40] Tanimoto I. M. F.; Cressiot B.; Greive S. J.; Pioufle B. L.; Bacri L.; Pelta J. Focus on Using Nanopore Technology for Societal Health, Environmental, and Energy Challenges. Nano Res. 2022, 1–15. 10.1007/s12274-022-4379-2. PMC9120803 35610982

[ref41] Meyer N.; Janot J.-M.; Lepoitevin M.; Smietana M.; Vasseur J.-J.; Torrent J.; Balme S. Machine Learning to Improve the Sensing of Biomolecules by Conical Track-Etched Nanopore. Biosensors 2020, 10 (10), 140 10.3390/bios10100140. 33028025 PMC7601669

[ref42] Reynaud L.; Bouchet-Spinelli A.; Janot J.-M.; Buhot A.; Balme S.; Raillon C. Discrimination of Α-Thrombin and Γ-Thrombin Using Aptamer-Functionalized Nanopore Sensing. Anal. Chem. 2021, 93 (22), 7889–7897. 10.1021/acs.analchem.1c00461. 34038092

[ref43] Zhang J.-H.; Liu X.-L.; Hu Z.-L.; Ying Y.-L.; Long Y.-T. Intelligent Identification of Multi-Level Nanopore Signatures for Accurate Detection of Cancer Biomarkers. Chem. Commun. 2017, 53 (73), 10176–10179. 10.1039/C7CC04745B. 28852755

[ref44] Yuan C.; Wu X.; Gao R.; Han X.; Liu Y.; Long Y.; Cui Y. Nanochannels of Covalent Organic Frameworks for Chiral Selective Transmembrane Transport of Amino Acids. J. Am. Chem. Soc. 2019, 141 (51), 20187–20197. 10.1021/jacs.9b10007. 31789030

[ref45] Guo Y.; Niu A.; Jian F.; Wang Y.; Yao F.; Wei Y.; Tian L.; Kang X. Metal-Organic Complex-Functionalized Protein Nanopore Sensor for Aromatic Amino Acids Chiral Recognition. Analyst 2017, 142 (7), 1048–1053. 10.1039/C7AN00097A. 28280809

[ref46] Ali M.; Nasir S.; Ensinger W. Stereoselective Detection of Amino Acids with Protein-Modified Single Asymmetric Nanopores. Electrochim. Acta 2016, 215, 231–237. 10.1016/j.electacta.2016.08.067.

[ref47] Schiopu I.; Iftemi S.; Luchian T. Nanopore Investigation of the Stereoselective Interactions between Cu2+ and d,l-Histidine Amino Acids Engineered into an Amyloidic Fragment Analogue. Langmuir 2015, 31 (1), 387–396. 10.1021/la504243r. 25479713

[ref48] Wang J.; Prajapati J. D.; Gao F.; Ying Y.-L.; Kleinekathöfer U.; Winterhalter M.; Long Y.-T. Identification of Single Amino Acid Chiral and Positional Isomers Using an Electrostatically Asymmetric Nanopore. J. Am. Chem. Soc. 2022, 144 (33), 15072–15078. 10.1021/jacs.2c03923. 35953064 PMC9413207

[ref49] Versloot R. C. A.; Arias-Orozco P.; Tadema M. J.; Lucas F. L. R.; Zhao X.; Marrink S. J.; Kuipers O. P.; Maglia G. Seeing the Invisibles: Detection of Peptide Enantiomers, Diastereomers, and Isobaric Ring Formation in Lanthipeptides Using Nanopores. J. Am. Chem. Soc. 2023, 145 (33), 18355–18365. 10.1021/jacs.3c04076. 37579582 PMC10450680

[ref50] Haensele E.; Banting L.; Whitley D. C.; Clark T. Conformation and Dynamics of 8-Arg-Vasopressin in Solution. J. Mol. Model. 2014, 20 (11), 2485 10.1007/s00894-014-2485-0. 25374389

[ref51] Christ-Crain M.; Gaisl O. Diabetes Insipidus. Press. Médicale 2021, 50 (4), 104093 10.1016/j.lpm.2021.104093. 34718110

[ref52] Manning M.; Balaspiri L.; Moehring J.; Haldar J.; Sawyer W. H. Synthesis and Some Pharmacological Properties of Deamino[4-Threonine,8-d-Arginine]Vasopressin and Deamino[8-d-Arginine]Vasopressin, Highly Potent and Specific Antidiuretic Peptides, and [8-d-Arginine]Vasopressin and Deamino-Arginine-Vasopressin. J. Med. Chem. 1976, 19 (6), 842–845. 10.1021/jm00228a023. 950656

[ref53] Saito M.; Tahara A.; Sugimoto T. 1-Desamino-8-d-Arginine Vasopressin (DDAVP) as an Agonist on V1b Vasopressin Receptor. Biochem. Pharmacol. 1997, 53 (11), 1711–1717. 10.1016/S0006-2952(97)00070-1. 9264324

[ref54] Lubecka E. A.; Sikorska E.; Sobolewski D.; Prahl A.; Slaninová J.; Ciarkowski J. Arginine-, d-Arginine-Vasopressin, and Their Inverso Analogues in Micellar and Liposomic Models of Cell Membrane: CD, NMR, and Molecular Dynamics Studies. Eur. Biophys. J. 2015, 44 (8), 727–743. 10.1007/s00249-015-1071-4. 26290060 PMC4628624

[ref55] Kasianowicz J. J.; Brandin E.; Branton D.; Deamer D. W. Characterization of Individual Polynucleotide Molecules Using a Membrane Channel. Proc. Natl. Acad. Sci. U.S.A. 1996, 93 (24), 13770–13773. 10.1073/pnas.93.24.13770. 8943010 PMC19421

[ref56] Brun L.; Pastoriza-Gallego M.; Oukhaled G.; Mathe J.; Bacri L.; Auvray L.; Pelta J. Dynamics of Polyelectrolyte Transport through a Protein Channel as a Function of Applied Voltage. Phys. Rev. Lett. 2008, 100 (15), 158302 10.1103/PhysRevLett.100.158302. 18518160

[ref57] Cressiot B.; Braselmann E.; Oukhaled A.; Elcock A. H.; Pelta J.; Clark P. L. Dynamics and Energy Contributions for Transport of Unfolded Pertactin through a Protein Nanopore. ACS Nano 2015, 9 (9), 9050–9061. 10.1021/acsnano.5b03053. 26302243 PMC4835817

[ref58] Bétermier F.; Cressiot B.; Muccio G. D.; Jarroux N.; Bacri L.; della Rocca B. M.; Chinappi M.; Pelta J.; Tarascon J.-M. Single-Sulfur Atom Discrimination of Polysulfides with a Protein Nanopore for Improved Batteries. Commun. Mater. 2020, 1 (1), 59 10.1038/s43246-020-00056-4.

[ref59] Robertson J. W. F.; Rodrigues C. G.; Stanford V. M.; Rubinson K. A.; Krasilnikov O. V.; Kasianowicz J. J. Single-Molecule Mass Spectrometry in Solution Using a Solitary Nanopore. Proc. Natl. Acad. Sci. U.S.A. 2007, 104 (20), 8207–8211. 10.1073/pnas.0611085104. 17494764 PMC1866312

[ref60] Haensele E.; Saleh N.; Read C. M.; Banting L.; Whitley D. C.; Clark T. Can Simulations and Modeling Decipher NMR Data for Conformational Equilibria? Arginine-Vasopressin. J. Chem. Inf. Model. 2016, 56 (9), 1798–1807. 10.1021/acs.jcim.6b00344. 27585313

[ref61] Jia W.; Hu C.; Wang Y.; Gu Y.; Qian G.; Du X.; Wang L.; Liu Y.; Cao J.; Zhang S.; et al. Programmable Nano-Reactors for Stochastic Sensing. Nat. Commun. 2021, 12 (1), 5811 10.1038/s41467-021-26054-9. 34608151 PMC8490433

[ref62] Du X.; Zhang S.; Wang L.; Wang Y.; Fan P.; Jia W.; Zhang P.; Huang S. Single-Molecule Interconversion between Chiral Configurations of Boronate Esters Observed in a Nanoreactor. ACS Nano 2023, 17 (3), 2881–2892. 10.1021/acsnano.2c11286. 36655995

[ref63] Bitchagno G. T. M.; Nchiozem-Ngnitedem V.-A.; Melchert D.; Fobofou S. A. Demystifying Racemic Natural Products in the Homochiral World. Nat. Rev. Chem. 2022, 6 (11), 806–822. 10.1038/s41570-022-00431-4. PMC9562063 36259059

[ref64] Oukhaled A.; Bacri L.; Pastoriza-Gallego M.; Betton J.-M.; Pelta J. Sensing Proteins through Nanopores: Fundamental to Applications. ACS Chem. Biol. 2012, 7 (12), 1935–1949. 10.1021/cb300449t. 23145870

[ref65] Pedregosa F.; Varoquaux G.; Gramfort A.; Michel V.; Thirion B.; Grisel O.; Blondel M.; Müller A.; Nothman J.; Louppe G.; et al. Scikit-Learn: Machine Learning in Python. arXiv 2012, 10.48550/arxiv.1201.0490.

[ref66] Lever J.; Krzywinski M.; Altman N. Principal Component Analysis. Nat. Methods 2017, 14 (7), 641–642. 10.1038/nmeth.4346.

[ref67] Hilbe J. M. International Encyclopedia of Statistical Science; Springer Berlin Heidelberg: Berlin, Heidelberg, 2011; pp 755–758.

