# Supplementary material for: Identification and Detection of a Peptide Biomarker and Its Enantiomer by Nanopore
Source: ACS Cent Sci. 2024 May 3;10(6):1167–78. doi: 10.1021/acscentsci.4c00020 (PMC11212137; doi:10.1021/acscentsci.4c00020)
Supplement: Supplementary file 1 — oc4c00020_si_001.pdf [file oc4c00020_si_001.pdf]

Identification and detection of a peptide biomarker and its enantiomer by nanopore

Laura Ratinho[1], Laurent Bacri[2], Bénédicte Thiebot*[1], Benjamin Cressiot*[1], Juan Pelta*[2]

[1] Université Paris-Saclay, Univ Evry, CY Cergy Paris Université, CNRS, LAMBE, 95000, Cergy, France

[2] Université Paris-Saclay, Univ Evry, CY Cergy Paris Université, CNRS, LAMBE, 91025, Evry-Courcouronnes, France

* Email: benedicte.thiebot@cyu.fr; benjamin.cressiot@cyu.fr; juan.pelta@univ-evry.fr

Table of content:

Supplementary information S1: Table of the blockade levels, dwell time and event analyzed in native and reducing conditions………………………………………………………… S2

Supplementary information S2: Representative histograms of the number of events as a function of the event duration ………………………………………………………………… S3

Supplementary information S3: Scatter plot representing the normalized average blockade level against the dwell time of each event independently or in a mix …………………………. S4

Supplementary information S4: Scatter plot representing the normalized average blockade level against the dwell time independently or in a mix in the presence of TCEP ………………... S5

Supplementary information S5: Arginine Vasopressin chemical representation of open and saddle conformations …………………..……………………………………………… S6

Supplementary information S6: Reducing conditions blank experiments ……………..……... S7

Supplementary information S7: Table of frequency for native and reduced conditions ……. S8

Supplementary information S8: Histograms of the normalized number of events of an equimolar mix of L- and D-AVP as a function of the applied voltage ……..…………………………... S9

Supplementary information S9: Histograms of the normalized number of events of an equimolar mix of L- and D-AVP as a function of salt concentration ………..………………………… S10

Supplementary information S10: Histograms of normalized blockade levels as a function of the normalized number of events and determination of most probable blockade levels at 110 mV in an equimolar mix ……….……………………………………………………………….. S11

Supplementary information S11: Table of the blockade levels and event analyzed in native and reducing mix conditions ……………………………………………………………… S12

Supplementary information S12: Histograms of normalized blockade levels as a function of the number of events and determination of most probable blockade level for L-AVP:D-AVP (25:75) ratio ……………………………………………………………………………... S13

Supplementary information S13: Principal Component Analysis to classify the D-AVP and L-AVP peptides ……..…………………………………………………………………….... S14

Supplementary information S14: Logistic regression to classify the blockades …………... S15

Supplementary information S15: No-equimolar L-AVP-D-AVP mixtures …….………… S16

Supplementary information S16: Purity analysis of L-AVP by mass spectrometry and liquid chromatography ………..……………………………………………………………….... S17

Supplementary information S17: Purity analysis of D-AVP by mass spectrometry and liquid chromatography ……………………………………..…………………………….... S18

	Blockade level		Number of events analyzed		Dwell Time		Number of events analyzed		Total number of events	
	50 mV	110 mV	50 mV	110 mV	50 mV	110 mV	50 mV	110 mV	50 mV	110 mV
L-AVP Type I	0.75 ± 0.01	0.72 ± 0.01	2099	2756	258 ± 25 µs	440 ± 32 µs	111	257	8398	11495
L-AVP Type IIa	0.43 ± 0.01	0.43 ± 0.01			740 ± 28 µs	817 ± 14 µs	741	1841		
L-AVP Type IIb	0.46 ± 0.01	0.48 ± 0.01								
D-AVP Type I	0.73 ± 0.01	0.71 ± 0.01	1461	3927	193 ± 13 µs	390 ± 62 µs	157	258	8222	9673
D-AVP Type IIa	0.40 ± 0.01	0.39 ± 0.01			891 ± 58 µs	1143 ± 33 µs	1227	3292		
D-AVP Type IIb	0.43 ± 0.01	0.43 ± 0.01								
L-AVP + TCEP	0.63 ± 0.01	0.53 ± 0.01	7849	13227	296 ± 10 µs	982 ± 15 µs	7318	12209	18319	17841
D-AVP + TCEP	0.58 ± 0.01	0.49 ± 0.01	5231	7523	386 ± 77 µs	1062 ± 11 µs	4519	7337	10546	10224

Supplementary information S1: ***Table of the blockade levels, dwell time and event analyzed in native and reducing conditions.***

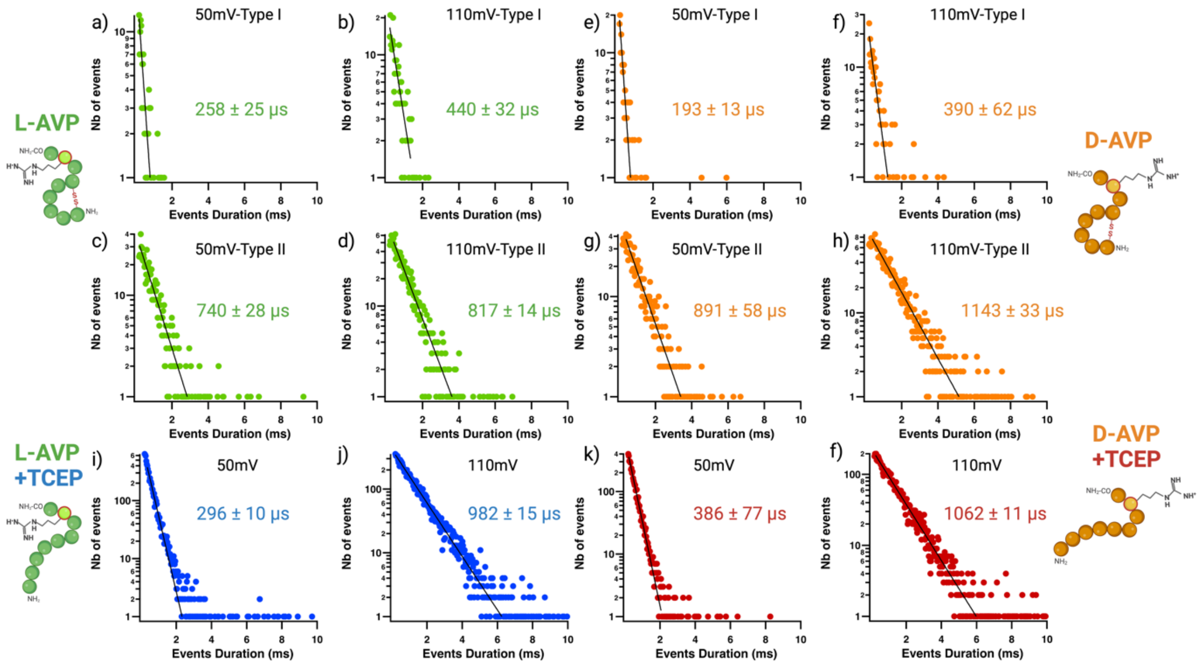

<u>Supplementary information S2:</u> Representative histograms of the number of events as a function of the event duration. *Representative histograms of the number of events as a function of the event duration fitted by an exponential. (a) Type I of events at 50 mV of L-AVP. Dwell time calculated = 258 ± 25 µs. (b) Type I of events at 110 mV of L-AVP. Dwell time calculated = 448 ± 32 µs. (c) Type II of events at 110 mV of L-AVP. Dwell time calculated = 740 ± 28 µs. (d) Type II of events at 110 mV of L-AVP. Dwell time calculated = 817 ± 14 µs. (e) Type I of events at 50 mV of D-AVP. Dwell time calculated = 193 ± 13 µs. (f) Type I of events at 110 mV of D-AVP. Dwell time calculated = 390 ± 62 µs. (g) Type II of events at 110 mV of D-AVP. Dwell time calculated = 891 ± 58 µs. (h) Type II of events at 110 mV of D-AVP. Dwell time calculated = 1143 ± 33 µs. (i) Dwell time calculated for L-AVP + TCEP at 50 mV = 296 ± 10 µs. (j) Dwell time calculated for L-AVP + TCEP at 110 mV = 982 ± 15 µs. (k) Dwell time calculated for D-AVP + TCEP at 50 mV = 386 ± 77 µs. (f) Dwell time calculated for D-AVP + TCEP at 110 mV = 1062 ± 11 µs. Data shown are from a single recording with fitted values being mean and standard deviation for three independent fits.* $N_{L\text{-}AVP\_TypeI\_50}$ *= 111 events;* $N_{L\text{-}AVP\_TypeI\_110}$ *= 257;* $N_{L\text{-}AVP\_TypeII\_50}$ *= 741 events;* $N_{L\text{-}AVP\_TypeII\_110}$ *= 1841 events;* $N_{D\text{-}AVP\_TypeI\_50}$ *= 147 events;* $N_{D\text{-}AVP\_TypeI\_110}$ *= 258;* $N_{D\text{-}AVP\_TypeII\_50}$ *= 1227 events;* $N_{D\text{-}AVP\_TypeII\_110}$ *= 3292 events;* $N_{L\text{-}AVP+TCEP\_50}$ *= 7318 events;* $N_{L\text{-}AVP+TCEP\_110}$ *= 12209;* $N_{D\text{-}AVP+TCEP\_50}$ *= 4519 events;* $N_{D\text{-}AVP+TCEP\_110}$ *= 7337 events. Dwell time and number of events were calculated by selecting each population with a dwell time superior to 200µs depending on their blockade level distribution. (Image generated using Biorender.com)*

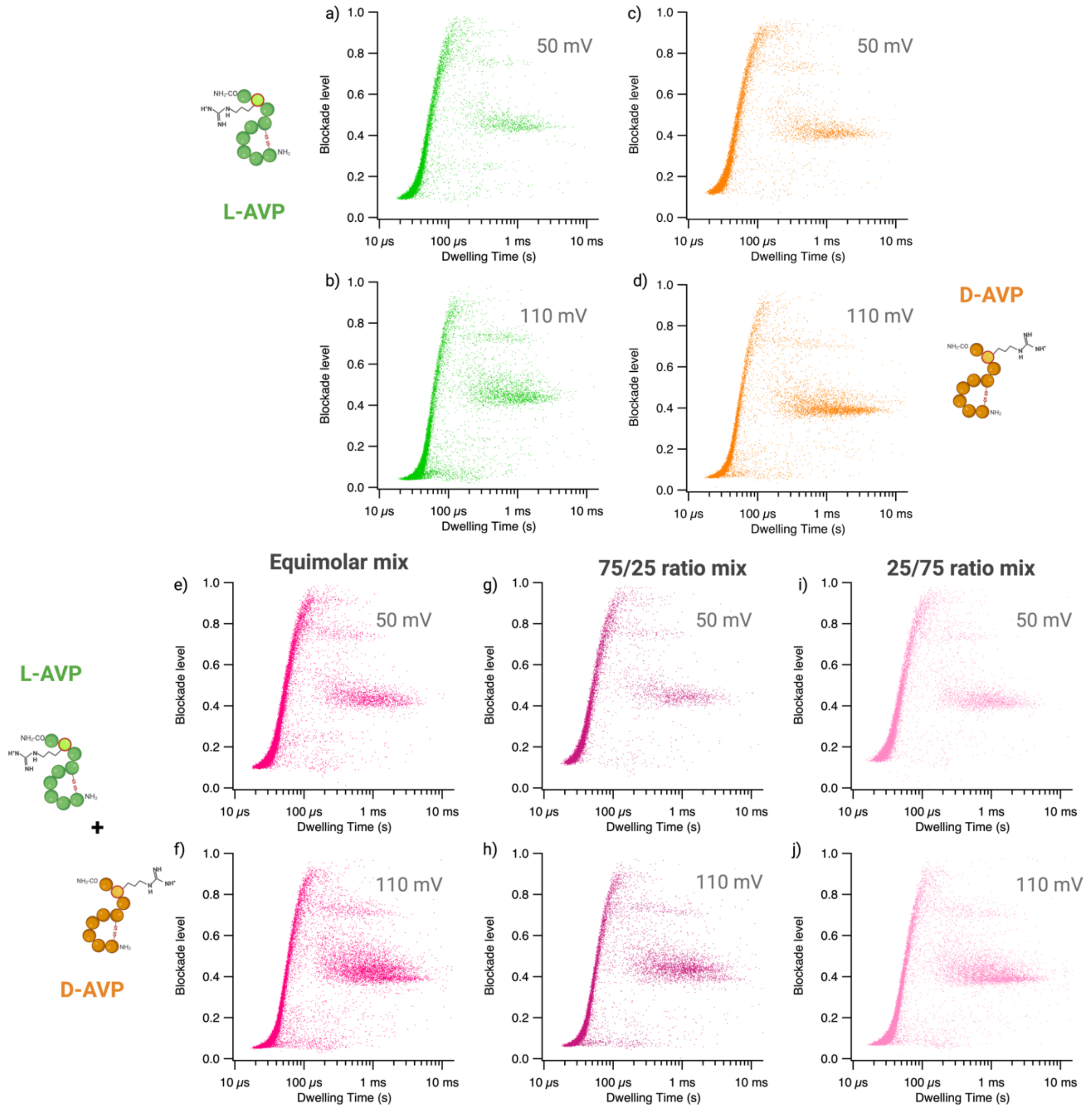

***<u>Supplementary information S3</u>*: Scatter plot representing the normalized average blockade level against the dwell time of each event independently or in a mix.** *Scatter plot representing the normalized average blockade level (DI$_b$) against the dwell time of each event between 0.0 and 1.0 in blockade level representing the interacting population of each peptide independently or in a mix. (a, c, e, g, i) Experiments recorded at 50 mV. (b, d, f, h, j) Experiments recorded at 110 mV. (a, b) 10 μM L-AVP. (c, d) 10 μM D-AVP. (e, f) Equimolar mix of 10 μM L- and D-AVP. (g, h) 7.5 μM L-AVP and 2.5 μM D-AVP. (i, j) 2.5 μM L-AVP and 7.5 μM D-AVP. Data shown are from a single recording. $N_{L\text{-}AVP\_50}$= 8398 events; $N_{L\text{-}AVP\_110}$= 11495; $N_{D\text{-}AVP\_50}$= 8222 events; $N_{D\text{-}AVP\_110}$= 9673 events; $N_{eq\text{-}mix\_50}$= 14114 events; $N_{eq\text{-}mix\_110}$= 16649 events; $N_{75/25\text{-}ratio\_50}$= 7448 events; $N_{75/25\text{-}ratio\_110}$= 10427 events; $N_{25/75\text{-}ratio\_50}$= 9022 events; $N_{25/75\text{-}ratio\_110}$= 14471 events. (Image generated using Biorender.com)*

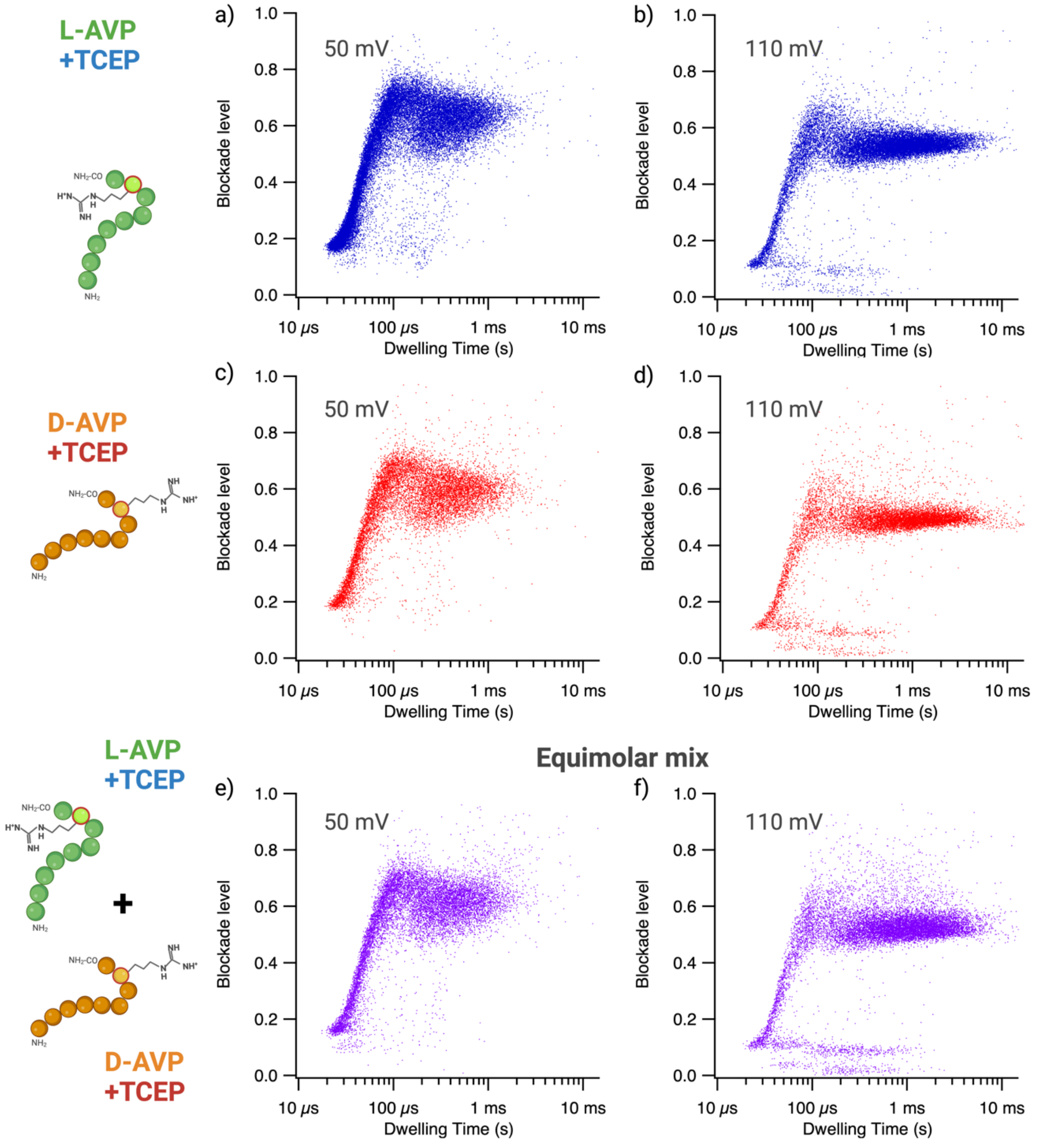

<u>Supplementary information S4:</u> Scatter plot representing the normalized average blockade level against the dwell time independently or in a mix in the presence of TCEP. *Scatter plot representing the normalized average blockade level (DI_b) against the dwell time of each event between 0.0 and 1.0 in blockade level representing the interacting population of each peptide independently or in a mix in the presence of TCEP. (a, c, e) Experiments recorded at 50 mV. (b, d, f) Experiments recorded at 110 mV. (a, b) 10 µM L-AVP, 5 mM TCEP. (c, d) 10 µM D-AVP, 5mM TCEP. (e, f) Equimolar mix of 5 µM L- and D-AVP with 5mM TCEP. Data shown are from a single recording. $N_{L\text{-}AVP+TCEP\_50}$= 26807 events; $N_{L\text{-}AVP+TCEP\_110}$= 26807; $N_{D\text{-}AVP+TCEP\_50}$= 10546 events; $N_{D\text{-}AVP+TCEP\_110}$= 10224 events; $N_{eq\text{-}mix+TCEP\_50}$= 18319 events; $N_{eq\text{-}mix+TCEP\_110}$= 17841 events. (Image generated using Biorender.com)*

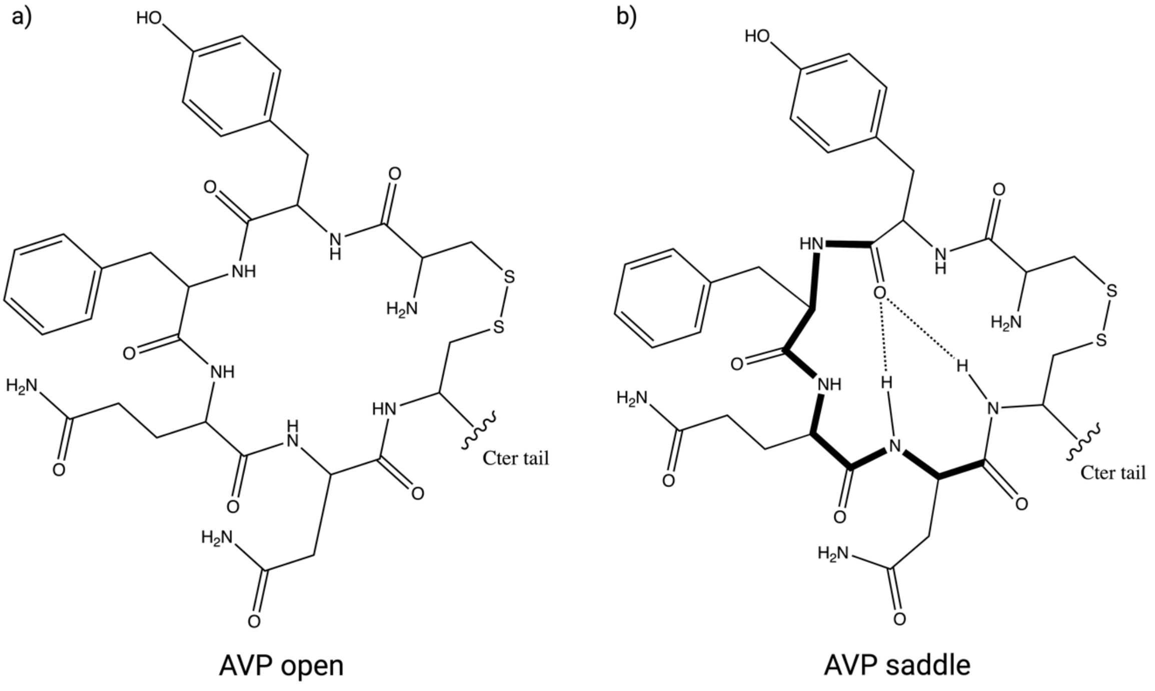

***<u>Supplementary information S5:</u>* Arginine Vasopressin chemical representation of open and saddle conformations.** *(a) the loop created by the disulfide bond take an open conformation. (b) Saddle conformation stabilized by two hydrogen bonds between C6 and Y2, N5 and Y2. (Chemical structure created with ChemDraw).*

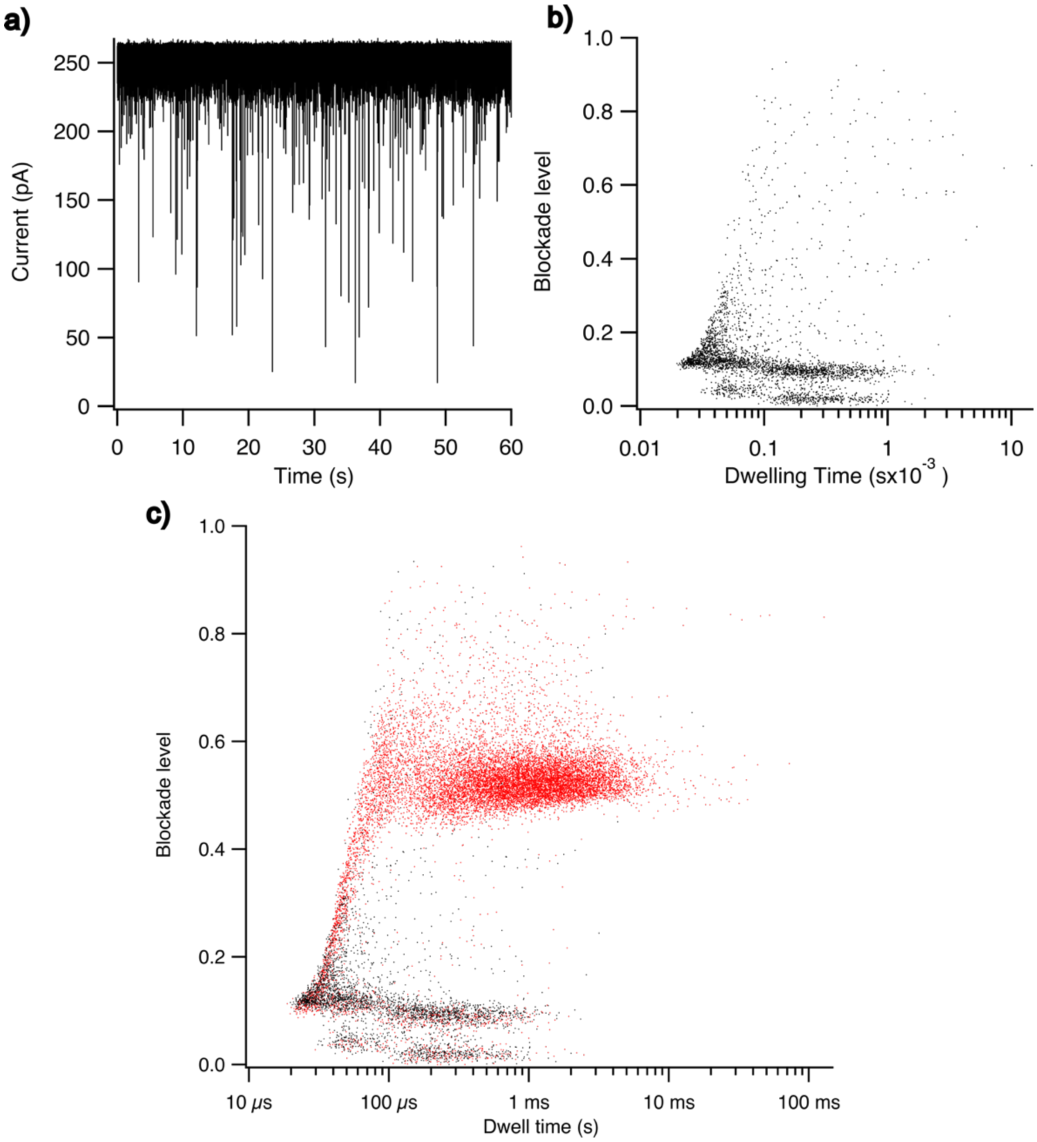

***<u>Supplementary information S6:</u>* Reducing conditions blank experiments**. *Blank TCEP 4M KCl, 25mM Tris HCl, pH 7.5, 5mM TCEP. (a) Current trace at 110 mV. Open pore current: I_0 = 252.21 +/- 3.90 pA. (b) Scatter plot representing the normalized average blockade level (DI_b) against the dwell time representing TCEP events. (c) Superposition of scatter plots representing the normalized average blockade level against the dwell time of the blank experiments (black) and the equimolar mix of 5μM L- and D-AVP + 5mM TCEP (red).*

	50 mV		110 mV	
	Global frequency	Population frequency	Global frequency	Population frequency
10μM L-AVP	46.1 ± 2.9 Hz	6.5 ± 0.2 Hz	100.8 ± 7.8 Hz	19.1 ± 1.0 Hz
10μM D-AVP	47.9 ± 0.8 Hz	8.9 ± 0.3 Hz	80.7 ± 1.1 Hz	28.1 ± 0.9 Hz
10μM L-AVP + 5mM TCEP	172.2 ± 4.1 Hz	65.5 ± 6.7 Hz	203.6 ± 14.4 Hz	153.3 ± 8.5 Hz
10μM D-AVP + 5mM TCEP	108.6 ± 6.3 Hz	55.4 ± 8.2 Hz	151.7 ± 11.9 Hz	107.2 ± 3.6 Hz

Supplementary information S7: Table of frequency for native and reduced conditions. *Table of frequency for each condition: 10μM L-AVP, 10 μM D-AVP, 10μM L-AVP+TCEP and 10μM D-AVP+TCEP. Data shown are from independent experiments.*

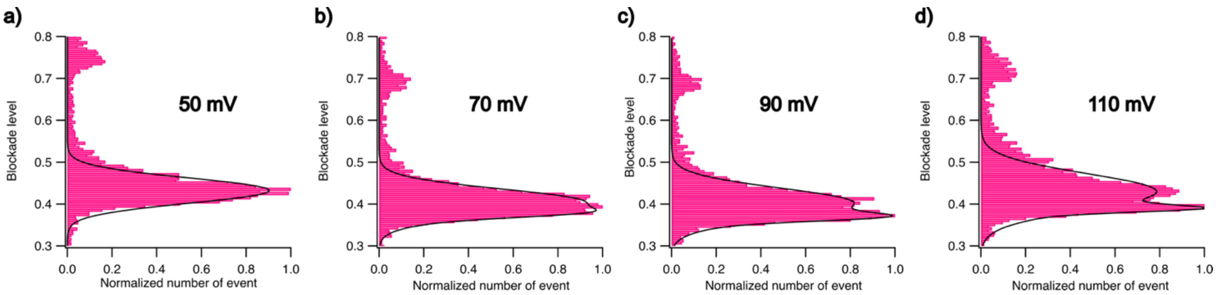

<u>Supplementary information S8:</u> Histograms of the normalized number of events of an equimolar mix of L- and D-AVP as a function of the applied voltage. *Histograms of the normalized number of events of 10µM equimolar mix of L- and D-AVP, depending on their blockade level at 50, 70, 90, and 110 mV, fitted by a bi-Gaussian. (a) Blockade level measured for 50mV: 0.43 +/- 0.01. (b) Blockade level measured for 70 mV: 0.38 +/- 0.01 et 0.40 +/- 0.01 representing the Type IIa most probable mean blockade level of L- and D-AVP at 70 mV. (c) Blockade level measured for 90 mV: 0.37 +/- 0.01 et 0.40 +/- 0.01 representing the Type IIa most probable mean blockade level of L- and D-AVP at 90 mV. (d) Blockade level measured for 110 mV: 0.39 +/- 0.01 et 0.43 +/- 0.01 representing the Type IIa most probable mean blockade level of L- and D-AVP at 110 mV. Data shown are from a single recording with fitted values being mean and standard deviation for three independent fits.* $N_{L\text{-}D\text{-}AVP\_eq\_50mV}$*= 3832 events;* $N_{L\text{-}D\text{-}AVP\_eq\_70mV}$*=4321 events;* $N_{L\text{-}D\text{-}AVP\_eq\_90mV}$*=4994 events;* $N_{L\text{-}D\text{-}AVP\_eq\_110mV}$*= 4630 events.*

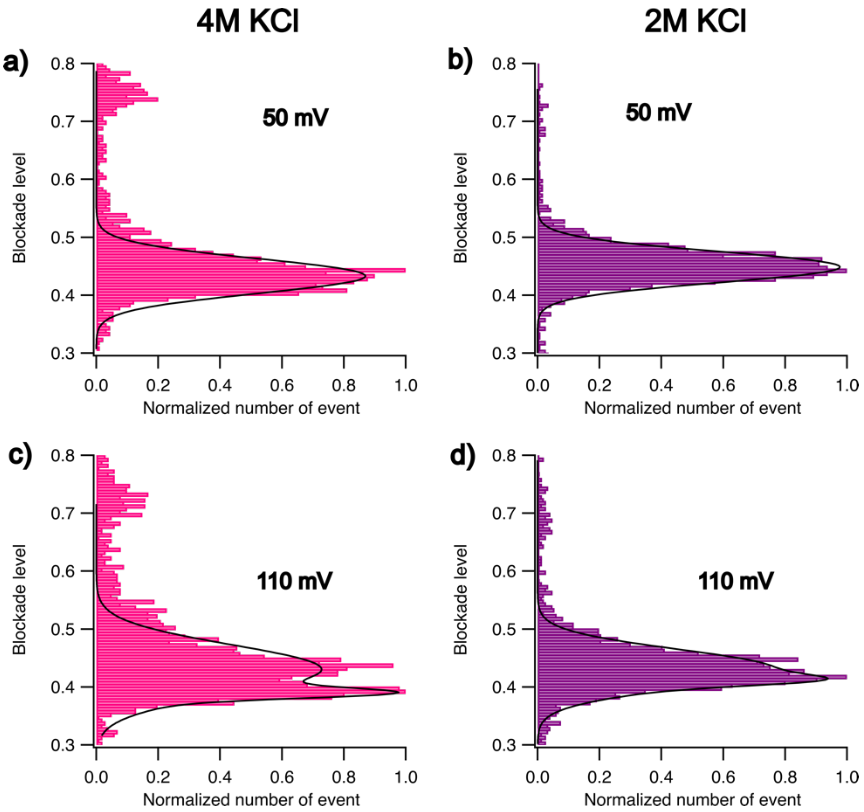

__Supplementary information S9:__* *Histograms of the normalized number of events of an equimolar mix of L- and D-AVP as a function of salt concentration. *Histograms of the normalized number of events of 10µM equimolar mix of L- and D-AVP, depending on their blockade level at 50 and 110 mV and salt concentration, fitted by a bi-Gaussian or Gaussian. (a, c) 4M KCl, 25mM Tris HCl, pH 7.4 (pink). (b, d) 2M KCl, 25mM Tris HCl, pH 7.4 (purple). (a, b) 50 mV. (c, d) 110 mV. (a) Blockade level measured: 0.43+/- 0.01. (b) Blockade level measured: 0.45+/- 0.01. (c) Blockade level measured: 0.39+/- 0.01 and 0.43 +/- 0.01, representing the Type IIa most probable mean blockade level of L- and D-AVP at 110 mV. (d) Blockade level measured: 0.41 +/- 0.01 and 0.43 +/- 0.01 representing the most probable mean blockade level of L- and D-AVP at 110 mV. Data shown are from a single recording with fitted values being mean and standard deviation for three independent fits.* $N_{L\text{-}D\text{-}AVP\_eq\_50mV\_4MKCl}$*= 2320 events;* $N_{L\text{-}D\text{-}AVP\_eq\_50mV\_2MKCl}$*=2500 events;* $N_{L\text{-}D\text{-}AVP\_eq\_110mV\_4MKCl}$*=1638 events;* $N_{L\text{-}D\text{-}AVP\_eq\_110mV\_2MKCl}$*= 1648 events.*

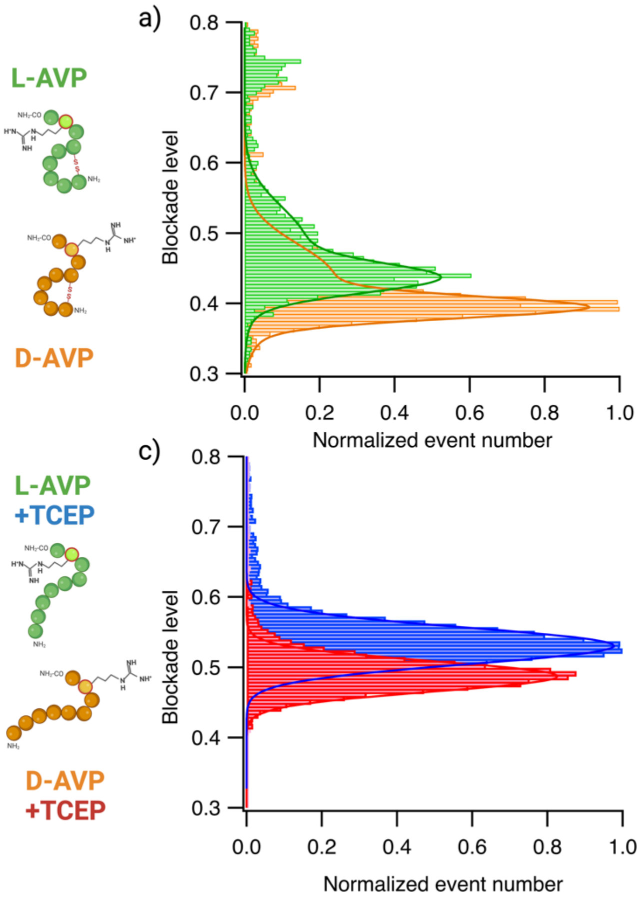

<u>Supplementary information S10:</u> Histograms of normalized blockade levels as a function of the normalized number of events and determination of most probable blockade levels at 110 mV in an equimolar mix. *Histograms of normalized blockade levels as a function of the normalized number of events fitted with a bi-Gaussian and/or a Gaussian to determine each population's most probable blockade levels at 110 mV. (a, c) Superposition of independent experiments of L-AVP (green) + D-AVP (orange) and L-AVP+TCEP (blue) + D-AVP+TCEP (red). (b, d) Addition of the independent histograms representing the expected results of equimolar mixes. (a) Type II blockade levels measured for L-AVP: Type IIa : 0.43 ± 0.01 and Type IIb : 0.48 ± 0.01; for D-AVP: Type IIa : 0.39 ± 0.01 and Type IIb : 0.43 ± 0.01. (b) Blockade levels measured: 0.43 ± 0.01 and 0.39 ± 0.01 representing the Type IIa most probable mean blockade level of L- and D-AVP at 110 mV. (c) Blockade levels measured for L-AVP + TCEP: 0.53 ± 0.01 and D-AVP + TCEP: 0.49 ± 0.01. (d) Blockade levels measured: 0.53± 0.01 and 0.48 ± 0.01 representing the mean blockade level of L- and D-AVP + TCEP at 110 mV. Data shown are from a single recording with fitted values being mean and standard deviation for three independent fits. $N_{L\text{-}AVP}$= 2156 events; $N_{D\text{-}AVP}$= 2822 events; $N_{L\text{-}AVP+TCEP}$= 10675 events; $N_{D\text{-}AVP+TCEP}$= 7552 events. Number of events were calculated by selecting the population with a dwell time superior to 200µs depending on their blockade level. (Image generated using Biorender.com)*

		Blockade level		Number of events analyzed		Total number of events	
		50 mV	110 mV	50 mV	110 mV	50 mV	110 mV
L-/D-AVP equimolar mix	L-AVP	0.43 ± 0.01	0.39 ± 0.01	3832	4630	14114	16649
	D-AVP		0.43 ± 0.01				
L-/D-AVP 75/25 ratio mix	L-AVP	0.44 ± 0.01	0.38 ± 0.01	3226	5907	7448	10427
	D-AVP		0.44 ± 0.01				
L-/D-AVP 25/75 ratio mix	L-AVP	0.42 ± 0.01	0.42 ± 0.01	1674	3884	9022	14471
	D-AVP		0.38 ± 0.01				
L-/D-AVP + TCEP equimolar mix	L-AVP	0.61 ± 0.01	0.50 ± 0.01	5460	4460	18319	17841
	D-AVP		0.52 ± 0.01				

Supplementary information S11: ***Table of the blockade levels and event analyzed in native and reducing mix conditions.***

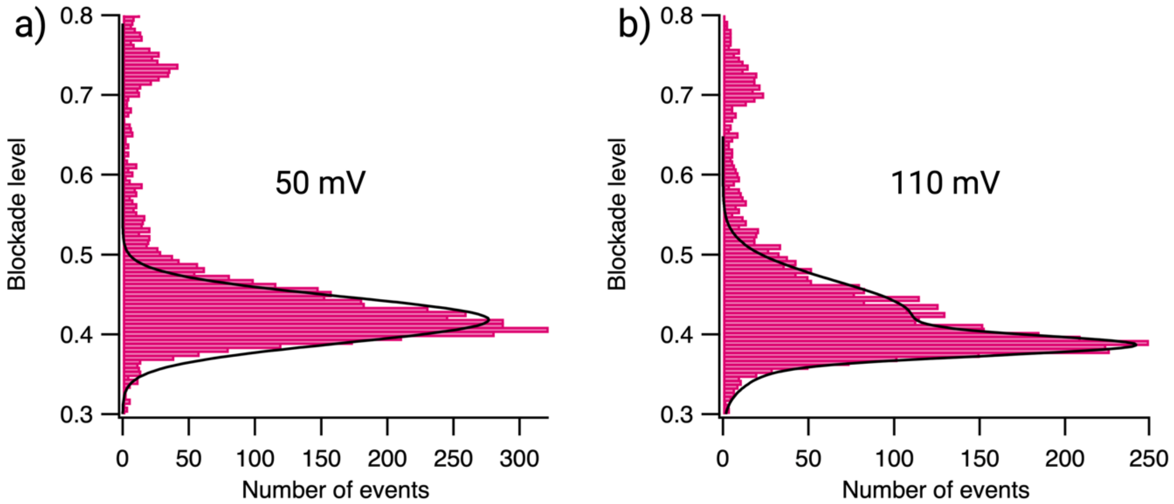

<u>Supplementary information S12:</u> Histograms of normalized blockade levels as a function of the number of events and determination of most probable blockade level for L-AVP:D-AVP (25:75) ratio. *Histograms of normalized blockade levels as a function of the number of events fitted with a bi-Gaussian and/or a Gaussian to determine each population's most probable blockade level of 2.5 µM L-AVP and 7.5 µM D-AVP in a mix. (a) Experiment recorded at 50 mV. Blockade level measured: 0.42 ± 0.01. (b) Blockade levels measured: 0.38 ± 0.01 and 0.42 ± 0.01 representing Type IIa most probable mean blockade level of L- and D-AVP in an equimolar mix at 110 mV. Data shown are from a single recording with fitted values being mean and standard deviation for three independent fits.* N_{50mV} *= 1674 events;* N_{110mV} *= 3884 events. Number of events were calculated by selecting the population with a dwell time superior to 200µs depending on their blockade level. (Image generated using Biorender.com)*

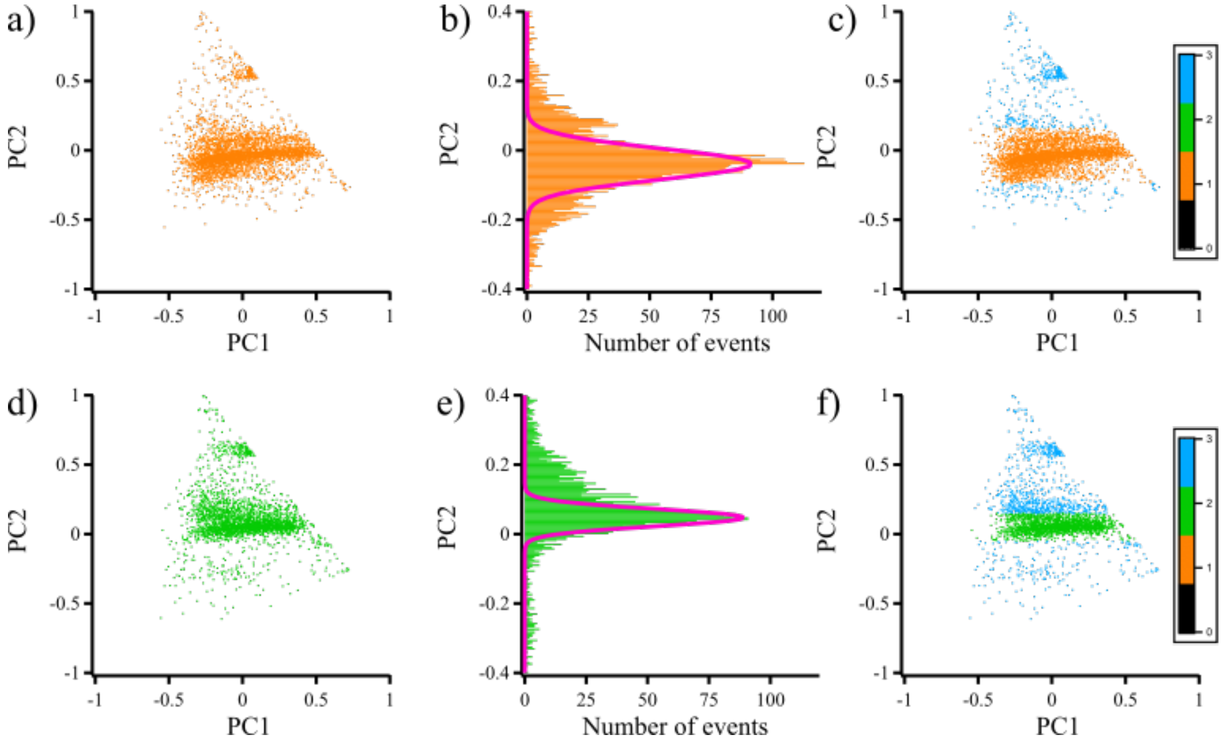

<u>Supplementary information S13:</u> *Principal Component Analysis to classify the D-AVP and L-AVP peptides.* *(a) Scatter plot of the two first principal components for D-AVP (b) Histogram of the second principal component. The curve in pink in a Gaussian fit (μ_D=-0.039, σ_D= 0.043). (c) PCA scatter plot. The blockades in orange belong to the range [μ_D-3·σ_D, μ_D+3·σ_D]. (d) Scatter plot of the two first principal components for D-AVP. (e) Histogram of the second principal component. The curve in pink in a Gaussian fit (μ_L=0.047, σ_L= 0.035). (f) PCA scatter plot. The blockades in orange belong to the range [μ_D-3·σ_L, μ_D+3·σ_L].*

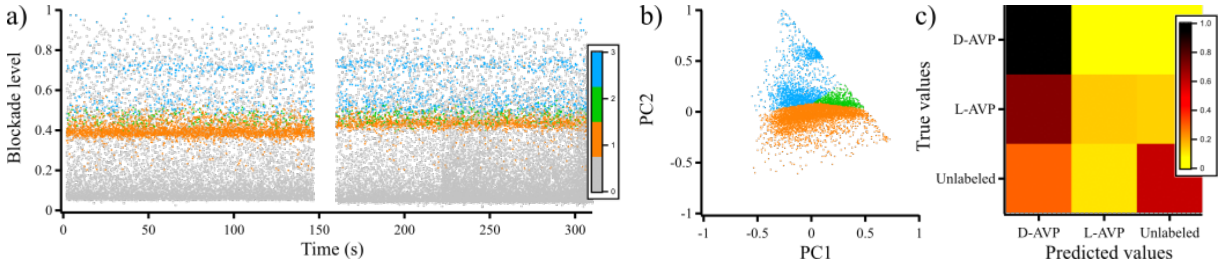

***<u>Supplementary information S14:</u>* Logistic regression to classify the blockades**. *(a) Blockade trace according to the 3 types of classification. The gray blockades correspond to the ones with standard deviation σ<1 pA. (b) Principal Component Analysis. Blockade classification according to 3 types (1: orange, 2: green, 3: blue). (c) Confusion matrix to evaluate the classification.*

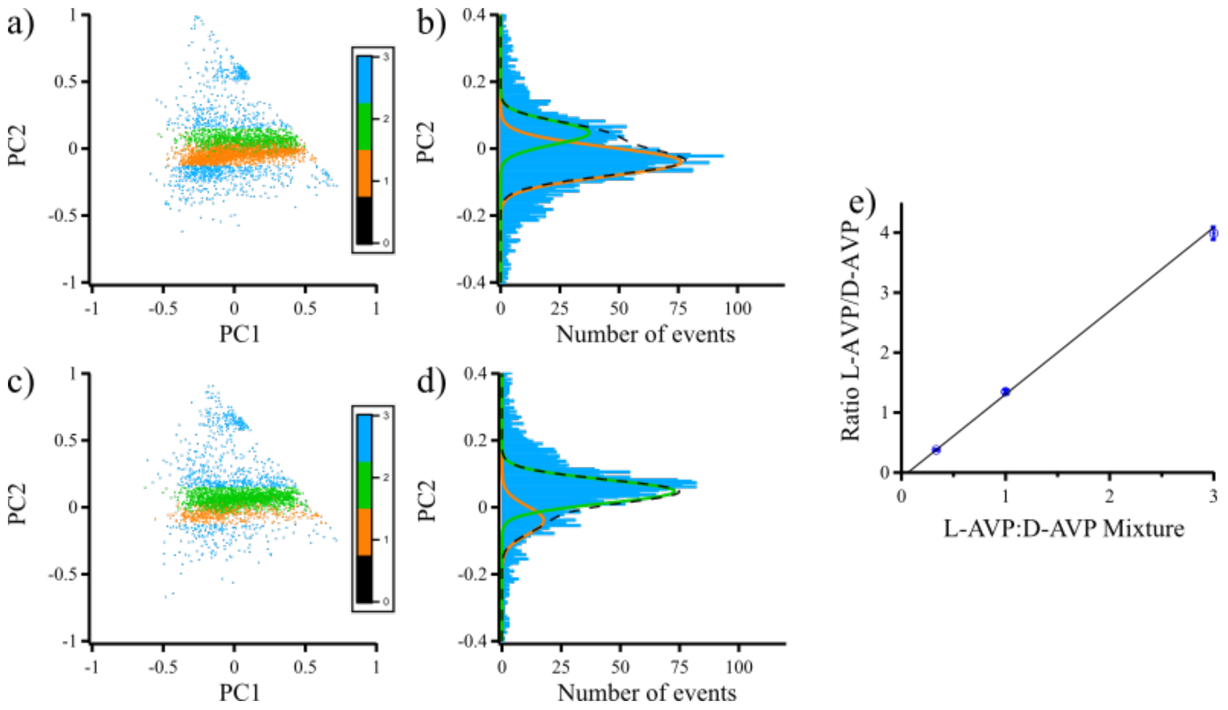

<u>Supplementary information S15:</u>* *No-equimolar L-AVP-D-AVP mixtures. *a) Scatter plot of the two first Principal Component Analysis for a 2.5µM L-AVP – 7.5µM D-AVP. Each data is labeled according to the type 1, type 2 (L-AVP), type 3(D-AVP). b) Distribution of the corresponding second Principal Component PC2. The histogram is fitted by 2 Gaussian functions (orange related to the D-AVP Type 1, green related to the L-AVP Type 2) and by the corresponding bi-Gaussian function (black dotted line). c) Scatter plot of the two first Principal Component Analysis for a 7.5µM L-AVP – 2.5µM D-AVP. Each data is labeled according to the type 1, type 2 (L-AVP), type 3(D-AVP). d) Distribution of the corresponding second Principal Component PC2. The histogram is fitted by 2 Gaussian functions (orange related to the D-AVP Type 1, green related to the L-AVP Type 2) and by the corresponding bi-Gaussian function (black dotted line). e) Ratio between the number of blockades for L-AVP and the ones for D-AVP according to the relative composition of the mixture L-AVP: D-AVP. The data are fitted by a line (slope 1.39).*

MASS SPECTROMETRY REPORT

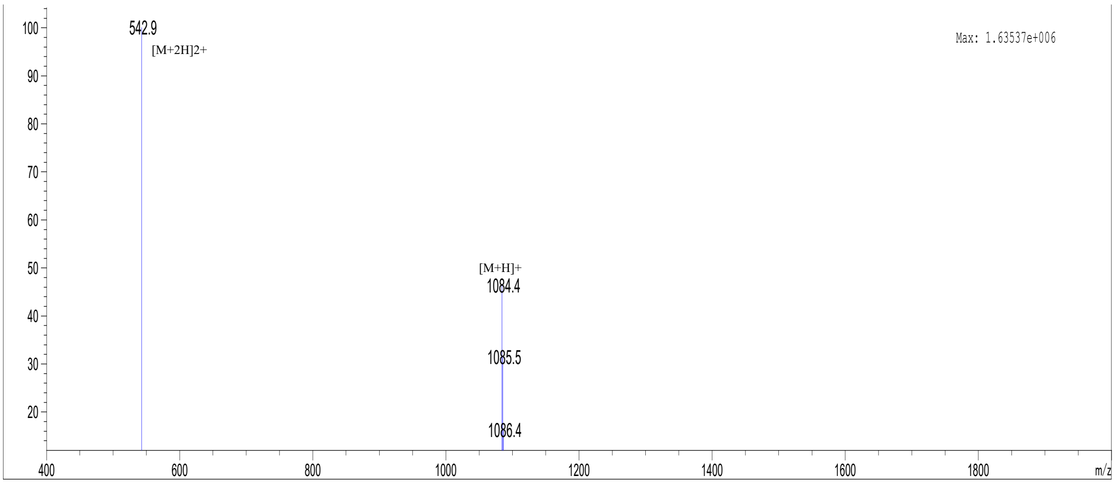

Sample Information		Probe:	ESI	Probe Bias:	+4.5kv
Injection Volume	: 1.00 μ l	Nebulizer Gas Flow:	1.5L/min	Detector:	1.5kv
Sample:	AVP L-Arg CG-9-NH2(O)	CDL:	-20.0v	T. Flow:	0.2ml/min
M.W.:	1084.24	CDL Temp.:	250 °C	B. Conc.:	50%H2O/50%ACN
Lot. No.:	P220416-SY983993	Block Temp.:	200 °C		

Sample: AVPL-Arg CG-9-NH2(0)
Lot. No.: P220416-SY983993
Column: Gemini-NX 5μ C18 110A, 4.6*250mm
Solvent A: A: 0.1% Trifluoroacetic Acid in 100% Acetonitrile
Solvent B: B: 0.1% Trifluoroacetic Acid in 100% Water
Gradient:

	A	B
0.0min	5%	95%
25.0min	30%	70%
25.1min	100%	0%
30.0min	Stop	

Volume: 10μl
Wavelength: 220nm
Flow rate: 1.0ml/min

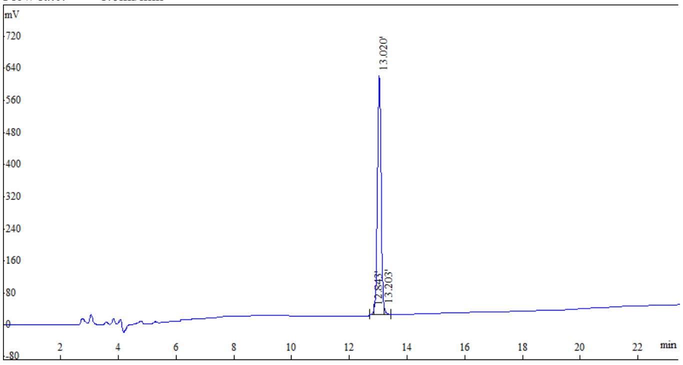

Rank	Time	Conc.	Area	Height
1	12.843	0.7495	36621	14441
2	13.020	98.2395	4800272	594109
3	13.203	1.0110	49402	15290
Total		100	4886295	623840

Supplementary information S16: *Purity analysis of L-AVP by mass spectrometry and liquid chromatography.*

MASS SPECTROMETRY REPORT

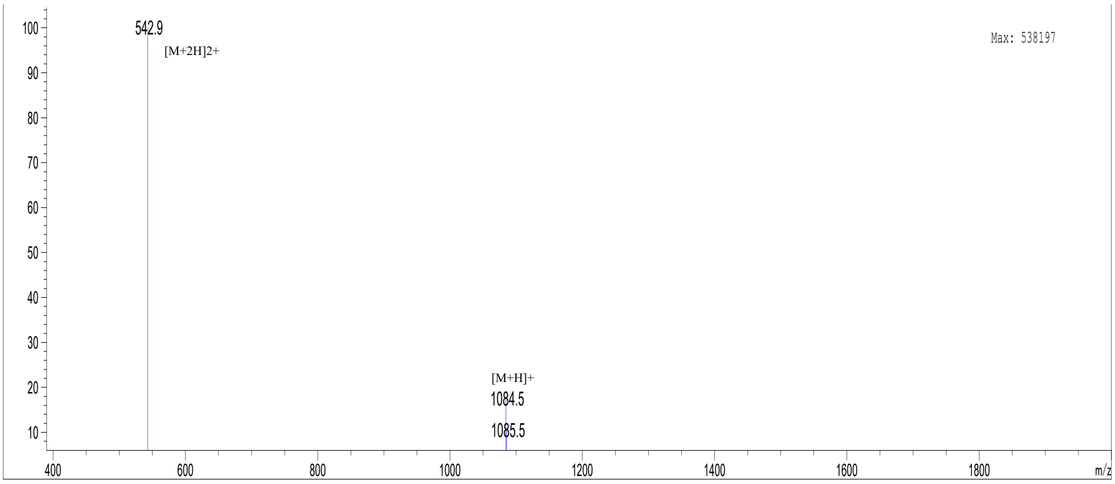

Sample Information		Probe:	ESI	Probe Bias:	＋4.5kv
Injection Volume :	1.00 μ l	Nebulizer Gas Flow:	1.5L/min	Detector:	1.5kv
Sample:	AVPD-Arg CG-9-NH2(O)	CDL:	-20.0v	T. Flow:	0.2ml/min
M.W.:	1084.24	CDL Temp.:	250 °C	B. Conc.:	50%H2O/50%ACN
Lot. No.:	P220413-SY983996	Block Temp.:	200 °C		

Sample: AVPD-Arg CG-9-NH2(0)
Lot. No.: P220413-SY983996
Column: Gemini-NX 5μ C18 110A, 4.6*250mm
Solvent A A: 0.1% Trifluoroacetic Acid in 100% Acetonitrile
Solvent B B: 0.1% Trifluoroacetic Acid in 100% Water
Gradient:

	A	B
0.0min	10%	90%
25.0min	35%	65%
25.1min	100%	0%
30.0min	Stop	

Volume: 10μl
Wavelength: 220nm
Flow rate: 1.0ml/min

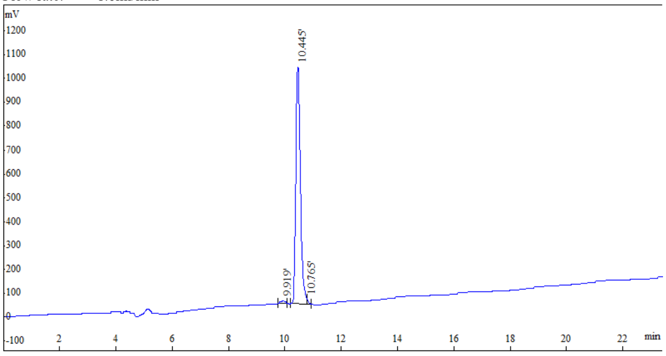

Rank	Time	Conc.	Area	Height
1	9.919	0.8144	87269	7718
2	10.445	98.5247	10557423	989992
3	10.765	0.6609	70815	20988
Total		100	10715507	1018698

Supplementary information S17: *Purity analysis of D-AVP by mass spectrometry and liquid chromatography.*
